# Analyses of 32 Loci Clarify Phylogenetic Relationships among *Trypanosoma cruzi* Lineages and Support a Single Hybridization prior to Human Contact

**DOI:** 10.1371/journal.pntd.0001272

**Published:** 2011-08-02

**Authors:** Carlos A. Flores-López, Carlos A. Machado

**Affiliations:** Department of Biology, University of Maryland, College Park, Maryland, United States of America; SBRI, United States America

## Abstract

**Background:**

The genetic diversity of *Trypanosoma cruzi,* the etiological agent of Chagas disease, has been traditionally divided in two major groups, *T. cruzi* I and II, corresponding to discrete typing units TcI and TcII-VI under a recently proposed nomenclature. The two major groups of *T. cruzi* seem to differ in important biological characteristics, and are thus thought to represent a natural division relevant for epidemiological studies and development of prophylaxis. To understand the potential connection between the different manifestations of Chagas disease and variability of *T. cruzi* strains, it is essential to have a correct reconstruction of the evolutionary history of *T. cruzi*.

**Methodology/Principal Findings:**

Nucleotide sequences from 32 unlinked loci (>26 Kilobases of aligned sequence) were used to reconstruct the evolutionary history of strains representing the known genetic variability of *T. cruzi*. Thorough phylogenetic analyses show that the original classification of *T. cruzi* in two major lineages does not reflect its evolutionary history and that there is only strong evidence for one major and recent hybridization event in the history of this species. Furthermore, estimates of divergence times using Bayesian methods show that current extant lineages of *T. cruzi* diverged very recently, within the last 3 million years, and that the major hybridization event leading to hybrid lineages TcV and TcVI occurred less than 1 million years ago, well before the contact of *T. cruzi* with humans in South America.

**Conclusions/Significance:**

The described phylogenetic relationships among the six major genetic subdivisions of *T. cruzi* should serve as guidelines for targeted epidemiological and prophylaxis studies. We suggest that it is important to reconsider conclusions from previous studies that have attempted to uncover important biological differences between the two originally defined major lineages of *T. cruzi* especially if those conclusions were obtained from single or few strains.

## Author Summary

*Trypanosoma cruzi* is the protozoan parasite that causes Chagas disease, a major health problem in Latin America. The genetic diversity of this parasite has been traditionally divided in two major groups: *T. cruzi I* and *II*, which can be further divided in six major genetic subdivisions (subgroups TcI-TcVI). *T. cruzi I* and *II* seem to differ in important biological characteristics, and are thought to represent a natural division relevant for epidemiological studies and development of prophylaxis. Having a correct reconstruction of the evolutionary history of *T. cruzi* is essential for understanding the potential connection between the genetic and phenotypic variability of *T. cruzi* with the different manifestations of Chagas disease. Here we present results from a comprehensive phylogenetic analysis of *T. cruzi* using more than 26 Kb of aligned sequence data. We show strong evidence that *T. cruzi* II (TcII-VI) is not a natural evolutionary group but a paraphyletic lineage and that all major lineages of *T. cruzi* evolved recently (<3 million years ago [mya]). Furthermore, the sequence data is consistent with one major hybridization event having occurred in this species recently (< 1 mya) but well before *T. cruzi* entered in contact with humans in South America.

## Introduction

*Trypanosoma cruzi* is the etiological agent of American Trypanosomiasis, also known as Chagas disease. Recent estimates suggest that about 15 million people in Latin America are infected with this parasite, and 12 to 20 thousand people die every year of the disease [1]. In nature, the parasite has two different cycles: a sylvatic cycle in which *T. cruzi* cycles between triatomines and wild mammalian reservoirs (e.g. opossums, raccoons, armadillos), and a domestic cycle in which *T. cruzi* infects humans through domiciliated triatomines [2], [3].

Since the 1980's the genetic variability and population structure of *T. cruzi* have been extensively characterized with a wide array of genetic markers [4], [5], [6], [7], [8], [9], [10], [11], [12], [13], [14]. Three main conclusions have been drawn from these studies: 1) *T. cruzi* has a mainly clonal mode of reproduction [5], [6], [12], although historical and experimental evidence of sporadic genetic exchange has been uncovered [9], [10], [15], [16], [17], [18],[19],[20],[21],[22],[23],[24]. 2) The genetic variability of *T. cruzi* can be divided in two major groups [7], [25], [26], [27], [28], [29], originally termed *T. cruzi* I and *T. cruzi* II [30]. *T. cruzi* II was additionally divided in 5 distinct subgroups or stable discrete typing units (DTUs IIa-IIe) [8], [31]. 3) DTUs IId and IIe are hybrids, the result of recent genetic exchange between ancestors of lineages IIb and IIc [10], [19]. Although a new intraspecific nomenclature was recently proposed [32], renaming the six major *T. cruzi* DTUs (I, IIa-IIe) as TcI-TcVI, no changes in the inferred division of *T. cruzi* in the two major evolutionary groups *T. cruzi* I (DTU TcI) and *T. cruzi* II (DTUs TcII-VI)) were implied or proposed.

The two major groups of *T. cruzi* seem to differ in important biological characteristics (e.g. pathogenicity in mice, doubling time of epimastigotes in vivo, susceptibility to drugs), and thus are thought to represent a natural division relevant for epidemiological studies and development of prophylaxis [33], [34], [35]. For instance, in the southern region of South America, where Chagas disease is most devastating, it has been observed that *T. cruzi* II strains (TcII-VI) are usually responsible for human infections, whereas *T. cruzi* I strains (TcI) are usually associated with the sylvatic cycle [36], [37], [38], [39], [40], [41]. Further, in regions north of the Amazon basin *T. cruzi* I strains are the main cause of Chagas disease, although the most acute manifestations of the disease are seemingly less common than in the southern cone of South America where most research on the disease has been conducted [22], [38], [42]. Thus, the current consensus is that *T. cruzi* II strains (TcII-VI) are more pathogenic to humans than *T. cruzi* I strains (TcI), although at least one author has clearly stated that the six DTUs (TcI-VI) should be considered the only relevant units of analyses for epidemiology and clinical studies [14].

Although the division of *T. cruzi* in two major evolutionary lineages has become deeply rooted in the literature, even leading to a recent suggestion that they correspond to two different species [43], there are strong reasons to doubt that this classification truly reflects the evolutionary history of this parasite. First, this classification is mostly based on codominant molecular markers (e.g. allozymes, microsatellites, RAPDs), which are not as phylogenetically informative as nucleotide sequences. Second, most studies that have used nucleotide sequences have not used an outgroup species in the phylogenetic reconstruction [11], [23], [44], [45]. That is a critical issue since the lack of outgroups does not allow for proper rooting of the tree and may lead to artificial evolutionary groupings. Further, with two exceptions [10], [46], the studies that have included outgroup sequences have failed to interpret the observed phylogenies in the context of the proposed division of *T. cruzi* in two major evolutionary groups. Third, in each of the few studies where outgroup sequences have been included, the two expected major monophyletic lineages corresponding to *T. cruzi* I (TcI) and II (TcII-VI) are not observed [10], [12], [18], [19], [46], [47], [48], [49]; instead, the evidence suggests that *T. cruzi* II (TcII-VI) is not a natural group since it appears to be paraphyletic.

To understand the diverse phenotypic differences among different *T. cruzi* strains and the potential connection between that variability and different manifestations of Chagas disease, it is essential to have a correct reconstruction of the evolutionary history of *T. cruzi*. A classification that represents evolutionary relationships is highly desirable because it may play an important role in strategic decisions about control and prophylaxis of Chagas disease. Here we present results from the largest sequence-based phylogenetic study of *T. cruzi* to date. We describe separate and combined phylogenetic analyses of nucleotide sequences from 31 nuclear genes and 1 mitochondrial region and provide estimates of the time of divergence of the main lineages of *T. cruzi*. We show that there is overwhelming evidence that *T. cruzi* II (TcII-VI) is not a natural evolutionary group but a paraphyletic lineage, and we provide a clear hypothesis of relationships among the six major DTUs of this parasite. Further, we estimate the time of diversification of *T. cruzi* strains and assess whether the sequence data is consistent with the two hybridization events that have been proposed for this species.

## Materials and Methods

### Samples

For every locus we collected sequences from *Trypanosoma cruzi* strains representing five of the six principal subgroups or discrete typing units (DTUs) of *T. cruzi*: TcI (I), TcIV (IIa), TcII (IIb), TcIII (IIc) and TcV (IId) (Table 1) [8]. Data from the sixth subgroup, TcVI (IIe), was already available as part of the *T. cruzi* genome sequence (www.genedb.org) [50]. Additional *T. cruzi* strains were sequenced in 9 of the 29 newly amplified loci (Tables 2 and S1). Sequences were also collected from two closely related bat trypanosomes, *T. cruzi marinkellei* (Strain N6) and *T. vespertilionis* (Strain 593), which were used as outgroups. All the strains used in this study have been widely characterized with a diverse array of genetic markers [6], [8], [9], [10], [18]. Purified DNA samples for all strains sequenced were provided by Michel Tibayrenc and Christian Barnabé from the Centre d'Etudes sur le Polymorphisme des Microorganismes (CEPM), CNRS (Montpellier, France).

**Table 1:** The main *Trypanosoma cruzi* strains used in this study.

Strain	DTU a	Zymodeme b	Isoenzyme type c	1999 nomenclature d	New nomenclature e
SO34 cl4	I	Z1	20	Tc I	TcI
SC13	I	Z1	?	Tc I	TcI
EP 255	IIa	Z3	nd(27)	Tc II	TcIV
CBB cl3	IIb	Z2	32	Tc II	TcII
M6241 cl6	IIc	Z2	35	Tc II	TcIII
SO3 cl5	IId	Z2	39	Tc II	TcV
CL Brener (CL F11F5)	IIe	Z2	43	Tc II	TcVI

a Discrete typing unit (DTU) [8];

b [4];

c [6];

d [30];

e [32].

**Table 2:** List of loci included in this study.

Locus ID	N a	bp b	Chromosome c	Gene location in Chr. (strand), gene length c	Predicted function	Topology d
COII-ND1	48	1226	Maxicircle (mtDNA)	N/A	Cytochrome oxidase subunit II-NADH dehydrogenase subunit 1	A
Tc00.1047053503555.30	11	1290	Chr 37	713055–714533 (−), 1479 bp	Trypanothione reductase (TR)	A
Tc00.1047053509153.90	40	1473	Chr 27	718463–720028 (+), 1566 bp	Dihydrofolate reductase-thymidylate synthase (DHFR-TS)	A
HSP70	11	508	Chr 32	699686–700540 (−), 855 bp	Intergenic region	A
Tc00.1047053503885.80	12	946	Chr 26	163788–164993 (+), 1206 bp	Hypothetical protein, conserved	A
Tc00.1047053503891.50	10	813	Chr 20	75320–76489 (−), 1170 bp	Hypothetical protein, conserved	A
Tc00.1047053503909.76	12	614		556434–557156 (+), 723 bp	Ferric reductase transmembrane protein, putative	B
Tc00.1047053504013.40	25	805	Chr 34	465693–466718 (−), 1026 bp	Serine acetyltransferase, putative	A
Tc00.1047053504045.100	10	886	Chr 40	1854961–1856415 (−), 1455 bp	Hypothetical protein, conserved	A
Tc00.1047053504057.80	10	858	Chr 34	417310–418677 (−), 1368 bp	Hypothetical protein, conserved	D
Tc00.1047053504059.20	13	896	Chr 14	465730–467526 (−), 1797 bp	Endomembrane protein, putative	A
Tc00.1047053506247.200	10	920	Chr 37	133811–136708 (+), 2898 bp	Beta-adaptin, putative	A
Tc00.1047053506525.150	13	821	Chr 40	593462–594415 (+), 954 bp	Hypothetical protein, conserved	A
Tc00.1047053506529.310	27	727	Chr 6	97318–98676 (−), 1359 bp	Hypothetical protein, conserved	C
Tc00.1047053506739.20	11	810	Chr 3	25655–27589 (−), 1935 bp	Hypothetical protein, conserved	F
Tc00.1047053507801.70	11	677	Chr 23	535126–535959 (+), 834 bp	Protein kinase, putative	A
Tc00.1047053508153.540	13	774	Chr 36	699363–700391 (+), 1029 bp	Hypothetical protein, conserved	A
Tc00.1047053508461.80	20	838	Chr 39	1187987–1189126 (−), 1140 bp	Prostaglandin F2alpha synthase	G
Tc00.1047053508719.70	24	709	Chr 37	375185–376402 (+), 1218 bp	Hypothetical protein, conserved	A
Tc00.1047053509007.30	13	815	Chr 31	573767–574690 (+), 924 bp	Hypothetical protein, conserved	E
Tc00.1047053509105.70	24	897	Chr 37	769449–770786 (−), 1338 bp	Thiol-dependent reductase 1, putative	A
Tc00.1047053509561.20	23	880	Chr 12	285842–287581 (−), 1740 bp	Flagellum-adhesion glycoprotein, putative	A
Tc00.1047053509967.50	11	595	Chr 10	184622–185329 (+), 708vbp	Hypothetical protein, conserved	A
Tc00.1047053510101.480	12	829	Chr 27	190063–191427 (−), 1365 bp	Hypothetical protein, conserved	B
Tc00.1047053510123.24	12	880	Chr 20	372476–373429 (+), 954 bp	Hypothetical protein, conserved	A
Tc00.1047053510131.90	12	936	Chr 30	340360–342003 (+), 1644 bp	Hypothetical protein, conserved	A
Tc00.1047053510765.50	13	817	Chr 39	1780396–1781763 (+), 1368 bp	Hypothetical protein, conserved	C
Tc00.1047053510877.190	8	453	Chr 34	493531–494328 (−), 798 bp	Hypothetical protein, conserved	A
Tc00.1047053510889.210	25	693	Chr 6	154383–156290 (−), 1908 bp	Hypothetical protein, conserved	A
Tc00.1047053510889.310	23	763	Chr 6	193929–196025 (+), 2097 bp	Hypothetical protein, conserved	A
Tc00.1047053511153.124	12	513	Chr 27	412720–413271 (+), 552 bp	Hypothetical protein, conserved	A
Tc00.1047053511529.200	13	667	Chr 35	170438–171232 (−), 795 bp	Hypothetical protein, conserved	A

a N: number of haplotypes sequenced.

b bp: sequenced region (in base pairs).

c [52].

d See Figure 1.

### Molecular methods

New sequence data was collected for 29 nuclear loci (Table 2). In addition, previously published data sets from one mitochondrial region (COII-ND1) and two nuclear genes (DHFR-TS, TR) [10], [18] were also included in the analyses, for a total of 32 loci. PCR primers were designed for 28 of the nuclear loci using Primer3 (Table S2) [51]; primers for the intergenic region of Hsp70 were previously published [20]. Loci were selected using the published genome sequence of the CL Brener strain of *Trypanosoma cruzi* [50]. Annotated loci were randomly selected from the genome based on two criteria: 1) lack of paralogous copies in the genome to avoid amplification of non-orthologous genes, 2) presence of conserved regions between both CL Brener haplotypes (if present) that would allow the design of conserved primers. The nuclear loci are located in 19 of the 41 predicted chromosomes of *T. cruzi* based on a recent genome assembly [52] (Table 2). Six of the 32 loci did not have a putative homolog in *T. brucei*. Putative function information for each locus was obtained from GeneDB and by conducting a blastp search on the *T. brucei* predicted protein database in GeneDB.

Conditions for the PCR amplifications were: 35 cycles of a 30 second denaturation step at 94°C, annealing at 56–60°C for 30 seconds, and extension at 72°C for 1 minute. PCR primers were used for bidirectional sequencing on a 3730xl DNA Analyzer (Applied Biosystems). Sequences were edited using Sequencher (GeneCodes). In cases where sequences had polymorphic nucleotides (determined by the presence of multiple double peaks in the chromatogram), PCR fragments were cloned using the TA cloning kit (Invitrogen) and three to five cloned PCR fragments were sequenced to identify both haplotypes. Singleton mutations that were observed only in the sequences from cloned fragments and not in the sequences from the PCR products were not included in the final sequence of each haplotype used in the analyses. Sequences have been deposited in GenBank (Accession Numbers HQ859465- HQ859886).

### Phylogenetic analyses

Sequences were manually aligned using SE-AL version 2.0 [53]. A Neighbor Joining (NJ) tree was reconstructed for each data set and each topology was used to estimate maximum likelihood parameters for different models of nucleotide substitution. The most appropriate nucleotide substitution model to analyze each locus was chosen using Modeltest 3.7 [54]. Maximum likelihood (ML) trees were individually obtained for each locus using ML heuristic searches in PAUP* 4.0b10 [55] using the tree bisection-reconnection (TBR) branch swapping algorithm. Bootstrap support values were obtained by ML analyses of 100 pseudoreplicates of each dataset.

MrBayes 3.1.2 [56], [57] was used to conduct Bayesian analyses using the substitution models chosen by Modeltest 3.7 [54]. We ran two independent simultaneous Markov Chain Mote Carlo runs with four chains each for 100,000 generations and sampled trees every 10 generations. If the standard deviation of split frequencies were not below 0.01 after analyses were done, the analyses were ran for an additional 100,000 generations and were stopped after convergence (i.e. standard deviation of split frequencies ≤ 0.01). Parameters and corresponding trees were summarized after discarding the initial 25% of each chain as burnin.

Data from the 32 loci were concatenated (26,329 nucleotides per strain) to reconstruct a consensus phylogenetic tree. Nuclear loci from the hybrid strains of *T. cruzi*, TcV (IId) and TcVI (IIe), usually have two different haplotypes, one of which groups with TcII (IIb) and the other with TcIII (IIc) [10], [18]. To analyze the concatenated data using haplotypes from the two hybrid strains included (SO3 cl5, CL Brener), we sorted each haplotype accordingly depending on the results from the ML and Bayesian phylogenetic analyses, concatenating haplotypes that had the same phylogenetic position (i.e. that grouped with the same “parental” clade). The concatenated alignment was analyzed using ML methods as described above. Bayesian analyses were performed in MrBayes 3.1.2 as described above, for 100 million generations with two parallel searches, with a burnin of 10% of the generations [56], [57].

**Figure 1:**
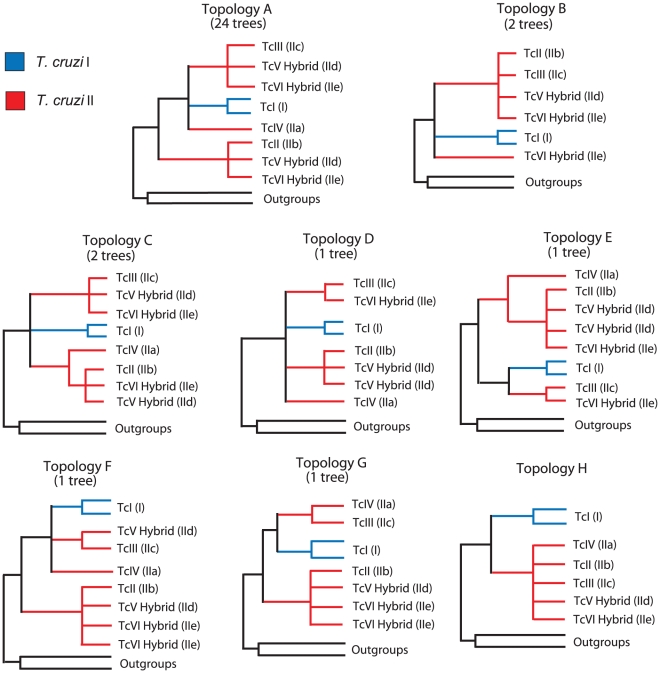
Phylogenetic topologies obtained from the 32 analyzed loci. Number on top of each topology represents the number of times that particular topology was observed (Table 2). All internal branches shown had bootstrap support values >70%. The topologies are depicted with respect to the classification system that divides *T. cruzi* in two major lineages [30], *T. cruzi I* (blue) and *T. cruzi II* (red), and the six major DTUs are labeled. Topology H is consistent with the current classification, and represents a history of divergence in which *T. cruzi I* and *II* are reciprocally monophyletic.

To test the topological congruence among the gene trees, we used PAUP* 4.0b10 [55] to perform the incongruence length difference test (ILD) among all data sets [58]. In addition, the Shimodaira-Hasegawa congruency test [59] was performed on each dataset as well as in the concatenated dataset in order to compare the likelihood of the phylogeny obtained by ML and the likelihood of the tree when *T. cruzi* I and II (TcI and TcII-VI) are enforced to be monophyletic (see Topology H, Figure 1). This was done in order to assess the support of the current division of *T. cruzi* in two major phylogenetic groups.

### Tests of selection

Non-neutral evolutionary patterns can affect inferences of phylogenetic relationships (e.g. [60]). Therefore each locus was examined for evidence of positive selection acting across the complete sequence and among codon sites using the codeml application from the PAML package [61]. Pairs of nested models were compared using a likelihood ratio test (LRT) under the assumption that the LRT statistic follows a chi-square distribution with the number of degrees of freedom dependent on the estimated number of parameters differentiating the nested models. We compared three pairs of nested site models: 1) M1 (neutral) versus M2 (selection); 2) M7 (beta) versus M8 (beta & ω); 3) M8 versus M8a (beta & ω  =  1) [62], [63]. Significance of the LRT of M1 vs M2 and M7 vs M8 was determined using 2 degrees of freedom. Since M8a is not fully nested on M8, a strict LRT for these two models is not possible. However, it has been suggested that significance of the LRT can be determined by halving the p value from a chi-square test with 1 degree of freedom [61].

### Divergence time estimates

Likelihood Ratio Tests (LRT) were performed to evaluate the null hypothesis that each locus of the concatenated dataset evolved under a molecular clock [64]. The molecular clock was rejected in only 3 genes (DHFR-TS, Tc00.1047053504059.20, Tc00.1047053509561.20) (Table S4). The remaining 22 loci in which the molecular clock was not rejected, and that had a homolog in *T. brucei*, were concatenated for these analyses. Divergence dates were estimated using Bayesian analysis in BEAST v1.5.3 [65]. Both the strict and relaxed Lognormal clock models were used to estimate divergence times on the mitochondrial and the concatenated nuclear loci data sets. Analyses were run separately for nuclear and mitochondrial sequences since previous analyses gave very different estimates for each type of data [10]. All analyses were conducted without any topological constraints using the HKY substitution model with the gamma plus invariant sites as the site heterogeneity model, with 4 gamma categories, as well as partitioning of codons into 3 positions. All priors were set to default values, except for the divergence estimate between *T. cruzi* and *T. brucei*, which was set to 100 million years ago (mya) under a normal distribution with 10 mya as the standard deviation. This date (100 mya) is a conservative estimate of the time to the last common ancestor of *T. cruzi* and *T. brucei* using the time of separation of Africa and South America [66]. Times of divergence were obtained by converging 10 independent Markov Chain Monte Carlo (MCMC) runs in Tracer v1.5 [65] in order to ensure convergence between the runs. Burnin of 20% of the samples was used. Each run had a chain length of 10 million, with sampling every 1000 chains. Although the mitochondrial data had been previously analyzed using a simpler method [10], we decided to reanalyze them with the Bayesian framework described above to compare previous estimates with the new Bayesian estimates.

The Relaxed Lognormal Clock model allows assessing how clock-like the data are (i.e. whether there is large rate heterogeneity among lineages), by using the estimate of the ucld.stedv parameter. A value of 0 means that the data is reasonably clock-like, whereas a value much greater than 1 indicates that the data has considerable rate heterogeneity among lineages [67]. The nuclear data set had a ucld.stdev of 0.392, while the mitochondrial data set had a higher ucld.stdev value (0.701), indicating higher rate heterogeneity among lineages. However, the Relaxed Lognormal Clock model for the mitochondrial data set did not converge even after combining 10 independent runs in Tracer. Therefore the estimates of the mitochondrial data with this model were not reliable and are not presented.

In addition, we analyzed *T. cruzi* genome sequence data [50] to obtain synonymous substitution (Ks) values for all annotated genes that had a single copy of each Esmeraldo-like (TcII (IIb)) and non-Esmeraldo-like (TcIII (IIc)) ortholog in the genome sequence. Our phylogenetic analyses (see below) show that nucleotide distances between Esmeraldo-like and non-Esmeraldo-like alleles from the heterozygous genome strain represent maximum distances within *T. cruzi*. Thus, those distances can be used to estimate the time to the most recent common ancestor of the major extant lineages of the parasite. A list of 4,568 Esmeraldo-like and non-Esmeraldo-like orthologs was obtained from Table S1 of El-Sayed et al [68] and sequences were downloaded from TriTrypDB (tritrypdb.org). The orthologous sequences were pairwise-aligned using ClustalW [69] and the resulting alignments were passed to PAML for estimation of Ks using the codeml program with the pairwise distance estimation option (runmode  =  -2) [61]. The average Ks value (0.0404) was used to estimate the time back to the most recent common ancestor of extant *T. cruzi* lineages using an estimate of the mutation rate for *T. brucei* [70],[71] (see Discussion).

## Results

### Phylogenetic analyses

The predominant clonal mode of propagation of *T.cruzi* and lack of evidence of intragenic recombination in the data (not shown) allow using nuclear gene sequences for reconstructing intraspecific phylogenies. The 31 nuclear loci we analyzed are randomly distributed in the genome. They are located in 19 of the 41 predicted chromosomes of *T. cruzi*, and when located on the same chromosome the loci are at least 30 Kb apart (in most cases >100 Kb apart) (Table 2). The ML and Bayesian phylogenetic analyses of each one of the 32 individual loci (Figure S1) produced seven different topologies (Figure 1). The ILD partition test confirmed that at least one of these trees was significantly different from the others (p = 0.01). All 32 loci confirm the paraphyletic nature of *T. cruzi* II. Analyses of 24 of the 32 loci produced individual phylogenetic trees with the same topology (topology A), including the three genes that we previously analyzed [10], [18] (Table 2). Sequences from *T. cruzi* II strains were never monophyletic in any of the genes surveyed (represented by Topology H). Topology A is consistent with a history of divergence in which *T. cruzi* II strains are paraphyletic.

To test the validity of the division of *T. cruzi* in two major groups, we performed the Shimodaira-Hasegawa test on each gene tree [59]. The test was conducted to determine if a constrained topology representing the division of *T. cruzi* into two different reciprocally monophyletic lineages, *T. cruzi* I (TcI) and *T. cruzi* II (TcII-VI), was as good an explanation of the data as the ML trees obtained for each gene. For every gene the constrained topologies in which *T. cruzi* I and *T. cruzi* II were reciprocally monophyletic were significantly worse than the ML phylogenies (Table S3), rejecting the prevalent idea that *T. cruzi* is divided in the two major evolutionary lineages *T. cruzi* I (TcI) and *T. cruzi* II (TcII-VI).

**Figure 2:**
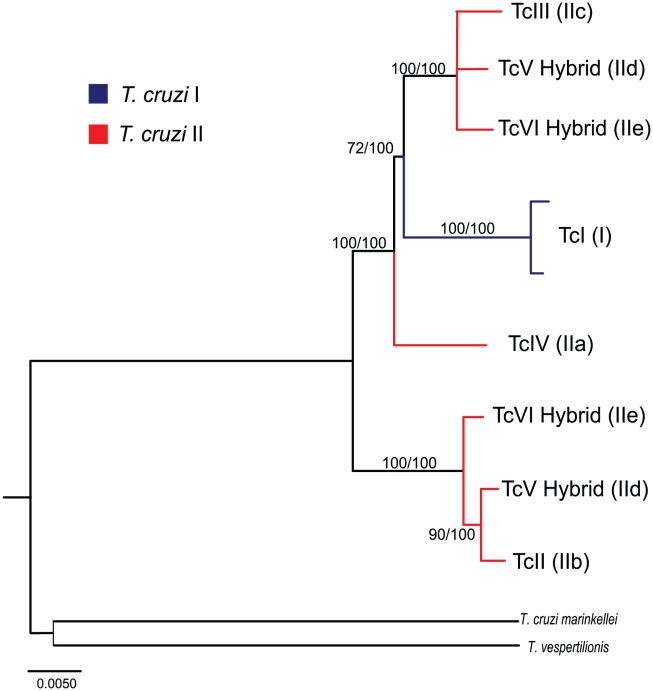
Maximum likelihood tree of concatenated data set. Data set consists of 31 nuclear loci and 1 mitochondrial region (COII-ND1), totaling 26,329 nucleotides per strain. Numbers above and below branches are Bootstrap (from ML analyses) and Bayesian support values, respectively. Taxon names represent the six major DTUs. Scale bar in number of substitutions per site.

ML and Bayesian phylogenetic trees reconstructed with the concatenated multilocus dataset (Figure 2) were also congruent with the ubiquitous topology A found on the majority of analyses of individual loci (Figure 1, Figure S1). All internal nodes in this topology are strongly supported either by ML or Bayesian analyses (Figure 2). Moreover, a constrained phylogeny consistent with the current division of *T. cruzi* in two major reciprocally monophyletic groups is significantly worse than the best ML tree from the multilocus concatenated dataset (p < 0.0001). This result provides further evidence that the current division of *T. cruzi* in two major evolutionary lineages [30] is a classification that does not reflect evolutionary relationships among strains of *T. cruzi*.

The basic relationships suggested by our analyses show that there are two major clades in the phylogeny of *T. cruzi.* The first clade, which harbors the most genetic diversity, includes DTUs TcI (I), TcIV (IIa), TcIII (IIc), and one haplotype from each of the two hybrid DTUs TcV (IId) and TcVI (IIe). The second lineage includes DTU TcII (IIb) and the other haplotype from each of the two hybrid DTUs TcV and TcVI. In 26 of the 32 nuclear loci analyzed we observed divergent allele sequences in members of both hybrid DTUs (TcV, TcVI) (Figure 1: Topologies A,C), in 4 loci both hybrid DTUs were homozygous or had barely divergent alleles (Figure 1: Topologies B,D,G), and in 2 loci one of the hybrid DTUs was homozygous while the other still had divergent alleles (Figure 1: Topologies E,F). Consistent with previous analyses [10], [19], we only observe evidence of one major hybridization event during the history of *T. cruzi*: between the ancestors of DTUs TcII and TcIII to generate DTUs TcV and TcVI (see Discussion).

### Selection tests

Only 8 of the 32 genes show evidence that some of their nucleotide sites have been under positive selection (Table 3). However, of these eight genes only four were highly significant in all three tests (M1 vs M2, M7 vs M8, M8 vs M8a). Three of the genes were only significant at the 5% level, but not at the 1% level, and only significant when M8a was compared to M8. The reconstructed phylogeny from 2 of the 8 genes that showed evidence of selection was different from the main topology A (Tc00.1047053506529.310: Topology C; Tc00.1047053510765.50: Topology C), but in none of those two cases sequences from all *T. cruzi* II strains were monophyletic. The other six genes that showed evidence of positive selection produced topology A. These results show that the loci used in this study are mostly evolving neutrally (24 out of 32 loci) and that phylogenetic analyses from 75% of the neutrally evolving loci (16 of 24) rendered the most common topology A (Figures 1 and 2), suggesting that results from the phylogenetic analyses have not been biased by loci that have been under positive selection.

**Table 3:** Results from the selection, distance and midpoint rooting analyses.

Locus ID	N	bp	% sites ω>1^a^	ω b	CL Brener ω c	Kimura d	Ks d	Midpoint rooting e
COII-ND1	48	1226	0	NA	Only 1 copy	0.095	0.3677	A
Tc00.1047053503555.30	11	1290	0	NA	0.08	0.016	0.0520	A
Tc00.1047053509153.90	40	1473	0.07	1.09	0.04	0.014	0.0403	A
Hsp70 f	11	508	NA	NA	NA	0.053	NA	A
Tc00.1047053503885.80	12	945	0.03	9.07***	1.47	0.038	0.0216	A
Tc00.1047053503891.50	10	810	0.06	6.17**	1.54	0.053	0.0268	A
Tc00.1047053503909.76	12	612	0.47	1.09	1.27	0.023	0.0231	B
Tc00.1047053504013.40	25	804	0	NA	0.37	0.029	0.0451	A
Tc00.1047053504045.100	10	885	0	NA	Only 1 copy	0.019	0.0428	A
Tc00.1047053504057.80	10	855	0	NA	Only 1 copy	0.015	0.0150	ND g
Tc00.1047053504059.20	13	894	0.02	2.88	0.74	0.019	0.0316	ND g
Tc00.1047053506247.200	10	918	0.03	2.67	Only 1 copy	0.018	0.0080	ND g
Tc00.1047053506525.150	13	819	0	NA	2.06	0.019	0.0049	A
Tc00.1047053506529.310	27	726	0.01	13.97***	0.42	0.028	0.0501	A g
Tc00.1047053506739.20	11	807	0.33	1.41	Only 1 copy	0.029	0.0120	B g
Tc00.1047053507801.70	11	675	0.03	4.79	∞	0.021	0.0002	ND g
Tc00.1047053508153.540	13	786	0.02	3.41	6.35	0.030	0.0036	H g
Tc00.1047053508461.80	20	699	0	NA	-	0.017	0.0388	G
Tc00.1047053508719.70	24	708	0.19	1.16	0.33	0.020	0.0292	H g
Tc00.1047053509007.30	13	813	0.58	1.25	1.59	0.028	0.0166	E
Tc00.1047053509105.70	24	849	0.07	4.07***	0.34	0.037	0.0531	H g
Tc00.1047053509561.20	23	879	0.11	5.62***	0.95	0.045	0.0318	A
Tc00.1047053509967.50	11	591	0	NA	2.22	0.024	0.0103	C g
Tc00.1047053510101.480	12	828	0.01	3.57	2.19	0.025	0.0109	G g
Tc00.1047053510123.24	12	879	0.09	3.21	3.76	0.037	0.0098	A
Tc00.1047053510131.90	12	933	0.01	2.54	2.81	0.022	0.0051	B g
Tc00.1047053510765.50	13	813	0.29	2.00*	1.58	0.025	0.0145	A g
Tc00.1047053510877.190	8	453	0.49	1.58	Only 1 copy	0.048	0.014	A
Tc00.1047053510889.210	25	693	0.02	5.96*	1.01	0.030	0.0234	A
Tc00.1047053510889.310	23	762	0.16	2.13	0.82	0.022	0.0343	A
Tc00.1047053511153.124	12	510	0.29	1.69	5.32	0.034	0.0063	C g
Tc00.1047053511529.200	13	666	0.008	20.94*	3.51	0.035	0.0089	H g

N: number of strains (haplotypes) sequenced. bp: number of aligned nucleotides used in the PAML analyses.

a The percent of sites with ω  =  dN/dS>1. ω estimated from the M8 model implemented in PAML. NA: Non-applicable, non-coding intergenic region.

b Average ω (dN/dS) for sites with dN/dS>1. NA: Non-applicable, since no sites had dN/dS>1.

c dN/dS estimated for the two haplotypes of CL Brener using PAML's codeml program with the pairwise distance estimation option (runmode  =  −2).

d Estimate of the % corrected distance for all sites (Kimura 2-parameter) or for synonymous sites only (Ks) between a strain of TcI (SC13) and a strain of TcII (CBB cl3), corresponding to the largest genetic distance within *T. cruzi*. NA: Non-applicable, non-coding intergenic region.

e The topology obtained with midpoint rooting (See Figure 1 for Topology definitions). ND (Non Described topology): the topology obtained was different from the topologies described in Figure 1.

f HSP70 is an intergenic region, thus selection tests were not conducted.

g The midpoint rooting topology was different from the topology reconstructed with an outgroup (Table 2).

*: Only significant for M8 vs M8a (p ≤ 0.05).

**: Significant for M8 vs M8a (p ≤ 0.01), M1 vs M2 (p ≤ 0.05), and M7 vs M8 (p ≤ 0.05).

***: p value ≤0.0001 in all three tests (M1 vs M2, M7 vs M8, M8 vs M8a).

### Estimates of divergence time

**Figure 3:**
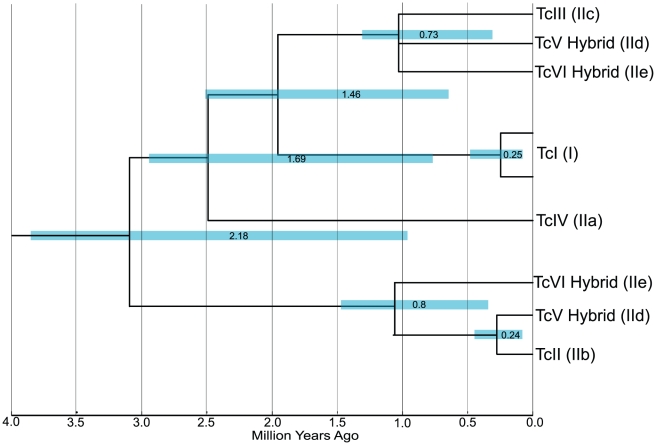
Divergence times for main DTU clades of *T. cruzi* using nuclear loci with the relaxed clock model. Data set consists of an alignment of 22 concatenated nuclear loci for which the molecular clock was not rejected (Table S4), and that had a homolog in *T. brucei*. Taxon names represent the six major DTUs. Scale bar in millions of years ago (mya).

The Molecular clock was rejected on the concatenated dataset (p<0.001). Therefore, each individual locus was tested for the molecular clock and loci for which a homolog could be confidently identified in *T. brucei* and for which the Likelihood Ratio Test could not reject the Molecular clock (21 loci, Table S4) were chosen to become part of a concatenated dataset suitable to run the Bayesian divergence time analyses. The divergence estimates from the mitochondrial dataset differ significantly from the nuclear loci estimates (Table 4). The estimated time to the most recent common ancestor (tMRCA) using mitochondrial data suggest that *T. cruzi*'s major lineages diverged during the Miocene (tMRCA  =  11.0 (7.0–15.2) mya), estimates that are similar to those presented by Machado and Ayala [10] using less sophisticated methods. On the other hand, the dates estimated with the concatenated data from 20 nuclear loci point towards a Pleistocene origin of *T. cruzi* (tMRCA  =  1.36 (1.0–1.7) mya (strict); tMRCA  =  2.18 (0.9–3.7) mya (relaxed)) (Table 4, Figures 3 and S2). Those dates are more recent than previously estimated divergence times using a single locus (TR: tMRCA  =  3.91 mya) [10]. We also obtained very similar divergence estimates from the concatenated data set of all nuclear loci that had a homolog in *T. brucei* (24 loci, Table S4) including genes that rejected the molecular clock hypothesis (not shown).

**Table 4:** Bayesian estimates of divergence time (in mya) for different *T. cruzi* lineages.

Nuclear loci (20 loci)							
Clockmodel	Tryps a	*T. cruzi* b	TcI c	TcI, TcIII-VI d	TcII-Hybrids f (TcV, TcVI)	TcIII-Hybrids f (TcV, TcVI)	PosteriorLikelihood
Strict	6.23(4.7–7.7)	1.36(1–1.7)	0.15(0.09–0.2)	1.11(0.8–1.4)	0.49(0.3–0.6)	0.49(0.3–0.6)	−57232.7477
Relaxedlognormal	8.3(3.8–13.8)	2.18(0.9–3.7)	0.25(0.08–0.5)	1.69(0.77–2.9)	0.8(0.3–1.4)	0.73(0.3–1.3)	−57183.9809

Mitochondrial loci (COII-ND1) h							
ClockModel	Tryps a	*T. cruzi* b	TcI c	TcI, TcIII-VI e	TcII g		PosteriorLikelihood
Strict	16.8(11.1–23.1)	11.00(7–15.2)	1.76(1–2.6)	7.5(4.7–10.5)	0.35(0.1–0.66)		−4670.0313

Times to the most recent common ancestor (tMRCA) are shown in mya. In parentheses are 95% HPD (highest posterior density) intervals.

a tMRCA of *T. cruzi* and its two outgroups (*T. c. marinkellei*, *T. verspertilionis*).

b tMRCA of extant *T. cruzi* lineages.

c tMRCA of TcI (nuclear data: SO34, SC13; mtDNA: TEH cl2, CEPA EP, Vin C6, X10 cl1, SABP3, A80, A92, MA-V, OPS21 cl11, CUTIA cl1, 133 79 cl7, V121, 26 79, CUICA cl1, SO34 cl4, P209 cl1, 85/818, P0AC, Esquilo cl1, SC13).

d tMRCA of strains SO34, SC13, CL35, EP225, CLA39-Haplotype1 and CL_Brener-Haplotype1.

e tMRCA of strains Florida C16, CANIII, M6241, CM 17, EP 255, 86-1, SO3, EPP, PSC-O, Tulahuen, CL F11F5, VM V4, P63, 86/2036, P251, X9/3, XII0/8 and XI09/2.

f tMRCA of TcII or TcIII and the respective closest haplotypes from both hybrid DTUs (TcV, TcVI).

g tMRCA of strains Esmeraldo, X-300, CBB, MCV, MSC2, TU18, MVB.

h The Relaxed Lognormal clock model for the mitochondrial data set did not converge even after combining 10 independent runs in Tracer. Therefore, the estimates from these analyses are not reliable and not shown here.

The discrepancy between the dates estimated with the mitochondrial and nuclear loci is likely the result of saturation of substitutions between the mitochondrial sequences of *T. cruzi* and the *T. brucei* outgroup used for the time calibration. Within *T. cruzi* the largest distance at silent sites (Ks) in the mitochondrial genes used is at least 6 times larger than that of any nuclear gene (Table 3), but most importantly substitutions at silent sites between *T. cruzi* and *T. brucei* are overly saturated (Ks  =  77.32). This observation is not surprising given the large divergence time between the two species, but leads to an overestimation of divergence times in more recently diverged lineages. For that reason we will not discuss the mitochondrial estimates any further.

The data allowed estimating the age of the major hybridization event in the history of *T. cruzi*: the generation of DTU's TcV and TcVI (IId and IIe) by hybridization of DTUs TcII and TcIII (IIc and IIb). The time of this event was estimated using the observed divergences between alleles from the putative parental and hybrid lineages (i.e. TcII vs TcV-TcVI and TcIII vs TcV-TcVI). This hybridization event occurred <1 mya, well before *T. cruzi* entered in contact with humans in South America, and the two independent estimates of the event are remarkably similar although the estimates from the strict clock model (tMRCA  =  0.49 (0.3–0.6) mya, 0.49 (0.3–0.6) mya) are more recent than the estimates from the relaxed lognormal clock model (tMRCA  =  0.8 (0.3–1.4) mya, 0.73 (0.3–1.3) mya) (Table 4, Figures 3 and S2).

## Discussion

### The evolutionary history of *Trypanosoma cruzi*

From the early 1990's *T. cruzi* was divided in two major groups, *T. cruzi* I and *T. cruzi* II [7], [25], [27], [28], [29], [30]. One of the groups, *T. cruzi* II, was further divided into 5 stable Discrete Typing Units (DTUs TcI-TcVI) based on additional genetic data [8], [31], [32]. Our study aims to clarify the phylogenetic relationships among the currently defined six major DTUs and represents a comprehensive molecular phylogenetic analysis of the largest nucleotide sequence dataset collected for this parasite (26,329 nucleotides per strain). Although we focused the sequencing on the seven strains listed in Table 1, for 10 of the 32 loci we obtained sequences from 20–48 strains (Tables 2 and S1). Results from the more deeply sampled loci are consistent with the overall results, and in particular there is no evidence of additional recombination/hybridization events (see below). The predominantly clonal population structure of *T. cruzi* [5], [6], [12] justifies sampling a limited number of strains representing the six major lineages of this parasite. The strains that constitute the core of the data presented here are widely studied standard laboratory strains which have been consistently used to make inferences about genetic and biological variability in *T. cruzi*. There is no indication that those strains represent outliers within *T. cruzi* and as such they are useful for making inferences about major evolutionary events in this parasite.

The concatenated phylogeny (Figure 2) is well supported and its topology is consistent with results from previous analyses of smaller sequence datasets that used outgroup sequences [10], [46]. Furthermore, it corresponds to the most commonly reconstructed topology using single loci (Topology A, Figure 1). This phylogeny shows that *T. cruzi* is divided in two clearly defined clades that do not correspond to the two originally defined major lineages *T. cruzi* I and *T. cruzi* II. Results from Shimodaira-Hasegawa tests applied to every locus (Table S3) provide strong evidence that the previously defined lineage *T. cruzi* II is paraphyletic and therefore does not represent a natural evolutionary lineage. One of the clades of the concatenated phylogeny includes TcI, TcIII, TcIV and one of the haplotypes from each of the two hybrid lineages TcV and TcVI. The other clade includes TcII and the alternative haplotypes from hybrid lineages TcV and TcVI. The phylogenetic placement of DTU TcIV (IIa) is less well resolved than the position of the other lineages. Although the bootstrap support of the branch separating TcIV from the TcI-TcIII-TcV-TcVI clade is 72% in the concatenated tree, the phylogenetic position of TcIV is quite variable in the individual trees (Figure S1). In 11 of the 24 trees consistent with Topology A (Figure 1) the placement of TcIV is the same as in the concatenated phylogeny and is supported with bootstrap values >55% (>80% in 5 trees). It is likely that the most sensible approach to attain full resolution of the phylogenetic position of TcIV is to increase the number of loci sampled. The availability of genome sequences of additional *T. cruzi* strains (e.g. [72]) should help resolve this issue.

Our results show that the classification of *T. cruzi* in two major evolutionary lineages [30], which has become deeply rooted in the literature, does not reflect the evolutionary history of this species. This classification arose from analyses of codominant molecular markers (e.g. allozymes, microsatellites, RAPDs) and PCR fragment sizes of different regions of rRNA genes and a mini-exon [7], [25], [26], [27], [28], [29], and appeared to be consistent with results from phylogenetic analyses of small nucleotide sequence datasets [11], [23], [44], [45]. However, none of those analyses included data from outgroups, a critical issue since lack of data from outgroup taxa does not allow for proper rooting of phylogenies and can generate artificial evolutionary groupings. Data from outgroups allow differentiating between derived (apomorphic) and ancestral (plesiomorphic) characters, which is fundamental for conducting proper phylogenetic analyses [73].

In our locus by locus analyses using outgroup data we never obtained topology H (Figure 1, Table 2), which corresponds to the phylogeny in which all *T. cruzi* II strains (TcII-VI) are monophyletic as suggested by the two group classification of *T. cruzi*. However, when we conducted the same analyses for every locus removing the outgroup sequences and rooting the tree at the longest internal branch (midpoint rooting), topology H was reconstructed 4 times (Table 3). Furthermore, in those analyses without outgroup we observed a different tree reconstructed in 15 of the 32 genes analyzed (Table 3). Those results suggest that the lack of outgroups in previous phylogenetic analyses of *T. cruzi* could be partially responsible for the original partition of the genetic diversity of this species in two major lineages.

The observation of distinct PCR fragment sizes in different regions of rRNA genes or mini-exon sequences [7], [27], [28], [29] was instrumental for the original division of *T. cruzi* in two major groups. Our phylogenetic results show that those studies simply uncovered derived character states in *T. cruzi* I (TcI) strains for the molecular traits studied, but the uncovered similarities in traits across strains do not correspond to actual evolutionary relationships among the strains. Presence-absence morphological or molecular characters can be useful for finding similarities among organisms but their utility for inferring evolutionary relationships is limited when the number of characters is very small and there is no additional supporting information. Without the context of a supported phylogeny it is not possible to determine if the observed character similarity truly reflects shared ancestry or homoplasy, as evidenced by the spurious relationships first described for *T. cruzi*.

### The age of Trypanosoma cruzi

Our calculations point towards a Pleistocene origin of the extant lineages of *T. cruzi* (tMRCA  =  1.36 (1.0–1.7) mya (strict); tMRCA  =  2.18 (0.9–3.7) mya (relaxed)) (Table 4, Figures 3 and S2). Furthermore, the major hybridization event that led to the origin of DTU's TcV and TcVI (IId and IIe) by hybridization of DTUs TcII and TcIII (IIc and IIb) occurred <1 mya, well before *T. cruzi* entered in contact with humans in South America. Estimated divergence times are dependent on the available calibration point(s), which in this study was the estimated separation time of Africa and South America (∼100 mya) based on geological evidence [66]. That date is thought to be the last time *T. cruzi* and *T. brucei* shared a common ancestor [74], [75]. Older divergence estimates for all the clades in the phylogeny can be obtained if older separation dates of Africa and South America are considered. However, obtaining estimates of *T. cruzi* divergence time as old as those suggested in other studies (e.g. 37–88 mya) [76], [77] requires using unrealistic calibration dates.

Even if there are uncertainties about the calibration point, the estimated recent divergence of *T. cruzi* is consistent with the small nucleotide divergences observed among the different lineages (Table 3) and leads to reasonable estimates of substitution rates in *T. cruzi*. The estimated silent site substitution rates per year (8.4–5.2×10^−9^) based on the average silent site divergence in *T. cruzi* (Ks  =  0.0228) and the estimated divergence times using nuclear loci (Table 4) fall within the range of silent site substitution rates estimated for other organisms [71], [78]. Further, independent estimates of the age of divergence of *T. cruzi* can be obtained using estimates of the nucleotide substitution rate per million year (my) and the observed average divergence at silent sites [79]. Using the estimated mutation rate in *T. brucei* (1.65×10^∼9^ per generation) [71] and its generation time (7–10 generations/year) [80], we obtain an estimate of the neutral mutation rate of 0.0115–0.0165 per my. Using that substitution rate and the observed average silent site divergence for 4569 single copy heterozygous genes from the *T. cruzi* genome (Ks  =  0.0404), the tMRCA of *T. cruzi* is estimated to be 1.73–1.21 mya, consistent with the phylogeny-based estimates obtained using BEAST (Table 4).

The recent divergence dates are also consistent with the idea that the diversification of *T. cruzi* was linked to the origin of its blood-sucking triatomine vectors, which occurred in the last 5 my [81], [82]. Molecular clock calibrations using *cytochrome b* sequences suggest a Pleistocene origin of *Rhodnius prolixus* and *R. robustus* [83], and the observation of almost identical transposable elements in *R. prolixus* and opossums and squirrel monkeys suggest a very recent association of vector and hosts [84].

### The evidence for hybridization events during *T. cruzi* divergence

Previous studies have established that hybridization events have played an important role during the diversification of this parasite [10], [11], [19], [23], [85]. Two different scenarios involving hybridization events have been proposed to explain the current genetic structure of *T. cruzi*. The first scenario proposes that a recent single hybridization event took place between the ancestors of DTU's TcII (IIb) and TcIII (IIc), which generated hybrid DTUs TcV (IId) and TcVI (IIe) [10], [11]. The second scenario proposes that in addition to the recent hybridization event responsible for hybrid DTUs TcV and TcVI, there was an ancestral hybridization event between the ancestors of DTUs TcI (I) and TcII that gave rise to the ancestors of DTUs TcIV (IIa) and TcIII [23], [85].

Our results provide additional evidence supporting the single recent hybridization event leading to the evolution of hybrid DTUs TcV (IId) and TcVI (IIe) [10], [11], [19]. The main evidence is the presence of multiple heterozygous loci with divergent alleles, where the alleles have close genetic distances to alleles from the putative parental lineages TcII (IIb) and TcIII (IIc). This pattern was first observed in several nuclear genes [10], [19] and later observed across thousands of genes in the genome sequence of *T. cruzi* strain CL Brener (TcVI) [50]. In this study we observed this pattern in 26 out of the 32 nuclear loci analyzed (Figure 1, Topologies A and C). More importantly, we did not observe any additional putative hybridization events that could be identified from loci with multiple polymorphic nucleotide sites. Our estimates of the age of the hybridization event suggest that this hybridization occurred less than 1 mya (Table 4, Figures 3 and S2), consistent with the observation that the alleles from the hybrid lineages have few nucleotide differences with the alleles from the putative parental lineages.

The ancestral hybridization event previously proposed [23], [85] requires the heterozygosity from the ancestral hybrid lineage to be lost through genome-wide homogenization by homologous recombination or gene conversion, given that the extant DTUs TcIV (IIa) and TcIII (IIc) show widespread homozygosity. This scenario suggests that the homogenization process should have left clear signals of the ancestral hybridization in patterns of SNP variation, which should show mixed signals of phylogenetic affinity to either one of the parental lineages. Unfortunately, two missing key factors in the original phylogenetic analyses conducted to support the ancestral hybridization event [23] have likely contributed to misinterpreting the data. The first and most important factor is the lack of outgroup sequences in the phylogenetic analyses. Our study shows that failure to include outgroup sequences can alter phylogenetic reconstruction in *T. cruzi* (Table 3). The second factor is the lack of bootstrap support values on key nodes of the trees that support the ancestral hybridization scenario.

We question the evidence for the ancestral hybridization scenario on three grounds. First, the origin of DTUs TcIII (IIc) and TcIV (IIa) is fairly recent, only about twice as old as the recent hybridization event leading to the origin of hybrid DTUs TcV (IId) and TcVI (IIe) (Table 4, Figures 3 and S2). It is therefore difficult to explain why there is still so much widespread allelic heterozygosity left in the hybrid DTUs TcV and TcVI, while there is (potentially) none left in DTUs TcIII and TcIV. For instance, the sequence of the genome strain CL Brener (TcVI) contains over 30 Mb of combined contig size in non-repetitive heterozygous regions and only 2 Mb in homozygous regions (see Table S2 from [50]). Given that pattern, it is clear that the proposed homogenization process that led to widespread loss of heterozygosity in the ancestor of DTUs TcIII and TcIV needs to be very different (at least in speed) than the process currently occurring in the recent hybrid strains. Second, the suggestion that DTUs TcIII and TcIV show mosaic sequences with SNPs that match DTUs TcI (I) or TcII (IIb) [23], [85] is hard to reconcile with patterns observed in our data, in data from a recent study [46], and in the sequenced strain of *T. cruzi*. To our knowledge there are no examples of obvious mosaic sequences in CL Brener, and, more importantly, the presence of interspersed SNPs matching either of the putative parental lines in small sequenced regions (∼1–2 Kb) will require fairly high rates of recombination which are not consistent with what is observed in the genome strain or in sequences from the hybrid strains. Third, a prediction of the ancestral hybridization scenario is that one should observe mixed phylogenetic signals across different loci [23]: in some loci, alleles from DTUs TcIV and TcIII will show strong phylogenetic affinities with alleles from DTU TcI, and in other loci with alleles from DTU TcII; other loci would show little phylogenetic resolution if they are mosaics from both ancestral parental lineages. Here, we have shown that there is overwhelming support (i.e. strong phylogenetic signal) linking alleles from DTUs TcIII and TcIV with alleles from DTU TcI (Figure 1, topology A), and in no case did we observe strong support for a link of DTUs TcIII and TcIV with alleles from DTU TcII (Figure 1, topology H; Table S3). To explain this pattern under the ancestral hybridization scenario one would also need to propose an additional mechanism whereby during homogenization there was gene conversion biased towards the allele from DTU TcI. Interestingly, the genome sequence of *T. cruzi* shows an excess of TcII-like homozygous regions relative to TcI-like homozygous regions (see Table S2 from [50]), contrary to the biased gene conversion towards TcI alleles required to explain our data under the ancestral hybridization scenario.

As the most appropriate explanation should be the most parsimonious, we suggest that the scenario requiring a single hybridization event leading to the generation of the extant hybrids DTUs TcV (IId) and TcVI (IIe) is the only one that is currently strongly supported by data. The analysis of complete genome sequences from multiple lineages of *T. cruzi* should provide a definitive test of the ancestral hybridization scenario, but it is telling that analyses of the large number of randomly selected loci presented here are not consistent with predictions from that hypothesis.

### Conclusion

We have reconstructed the evolutionary history of the major lineages of the human parasite *Trypanosoma cruzi* using nucleotide sequences from one mitochondrial region and 31 unlinked nuclear loci. Our results show that the original classification of *T. cruzi* in two major groups, *T. cruzi I* (TcI) and *T. cruzi II* (TcII-VI), does not reflect the evolutionary history of the parasite, that its diversification into the current extant lineages was recent (<1–3 mya), and that there is only strong evidence for one major hybridization event that occurred <1 mya, well before *T. cruzi* entered in contact with humans in South America. It is possible that by sampling a small number of strains one could miss detecting rare recombination or hybridization events (although we did not see this in loci that were more deeply sampled). Thus, future multilocus phylogenetic studies should also attempt conducting more in-depth sampling of strains. Based on our results we suggest that it is important to reconsider conclusions from previous studies that have attempted to uncover important biological differences between the two originally defined major lineages of *T. cruzi*. Conclusions from studies that report results of analyses from one or few strains that do not encompass all the genetic variability of the artificial group “*T. cruzi* II” should be carefully dissected to determine if the findings do in fact reflect fundamental biological differences between the natural group “T. cruzi I” and the artificial group “T. cruzi II” or simply reflect differences among the specific DTUs studied. A thorough review of the literature suggests that many of the studies that report differences, or lack thereof, between the two originally defined lineages of this parasite are typically based on observations from very few strains (Flores-López and Machado, in prep.). Future work should focus on trying to determine if, as previously suggested [14], the currently defined six major lineages of this parasite (TcI-TcVI), for which we now have well supported evolutionary relationships, do indeed represent independent relevant groups for epidemiological studies and development of prophylaxis.

## Supporting Information

Figure S1 **Individual Maximum likelihood trees of each amplified locus.** The most appropriate substitution model to analyze each locus was chosen using Modeltest 3.7 [54]. All trees were obtained for each locus using ML heuristic searches in PAUP* 4.0b10 using the tree bisection-reconnection (TBR) branch swapping algorithm. Bootstrap support values were obtained by ML analyses of 100 pseudoreplicates of each dataset. Strains (codes used in the figure): M6241 cl6 (CL35), CL Brener (Genome), SO3 cl5 (CLA39), EP255, SC13, SO34 (SO34 cl4), CBB cl3 (CBB32).
(PDF)

Figure S2 **Divergence times for main DTU clades of** ***T. cruzi*** **using nuclear loci with the strict clock model.** Data set consists of aligned concatenated nuclear loci (22) for which the molecular clock was not rejected (Table S4), and had a homolog in *T. brucei*. Codes (Strains): TcI (SO34 cl4 & SC13), TcIV (EP 255), TcII (CBB cl3), TcIII (M6241 cl6), TcV Hybrid (SO3 cl5), TcVI Hybrid (CL Brener) (See Table 1). Scale bar in millions of years ago.
(PDF)

Table S1 **Additional *Trypanosoma cruzi* strains used in this study.**
(DOC)

Table S2 **Amplified loci and PCR primers.**
(DOC)

Table S3 **Results of the Shimodaira-Hasegawa tests.**
(DOC)

Table S4 **LRT of Molecular clock on genes that had a homolog in *T. brucei.***
(DOC)
