# Supplementary material for: Analyses of 32 Loci Clarify Phylogenetic Relationships among Trypanosoma cruzi Lineages and Support a Single Hybridization prior to Human Contact
Source: PLoS Negl Trop Dis. 2011 Aug 2;5(8):e1272. doi: 10.1371/journal.pntd.0001272 (PMC3149036; doi:10.1371/journal.pntd.0001272)
Supplement: Figure S1 — Individual Maximum likelihood trees of each amplified locus. The most appropriate substitution model to analyze each locus was chosen using Modeltest 3.7 [54]. All trees were obtained for each locus using ML heuristic searches in PAUP* 4.0b10 using the tree bisection-reconnection (TBR) branch swapping algorithm. Bootstrap support values were obtained by ML analyses of 100 pseudoreplicates of each dataset. Strains (codes used in the figure): M6241 cl6 (CL35), CL Brener (Genome), SO3 cl5 (CLA39), EP255, SC13, SO34 (SO34 cl4), CBB cl3 (CBB32). (PDF) [file pntd.0001272.s001.pdf]

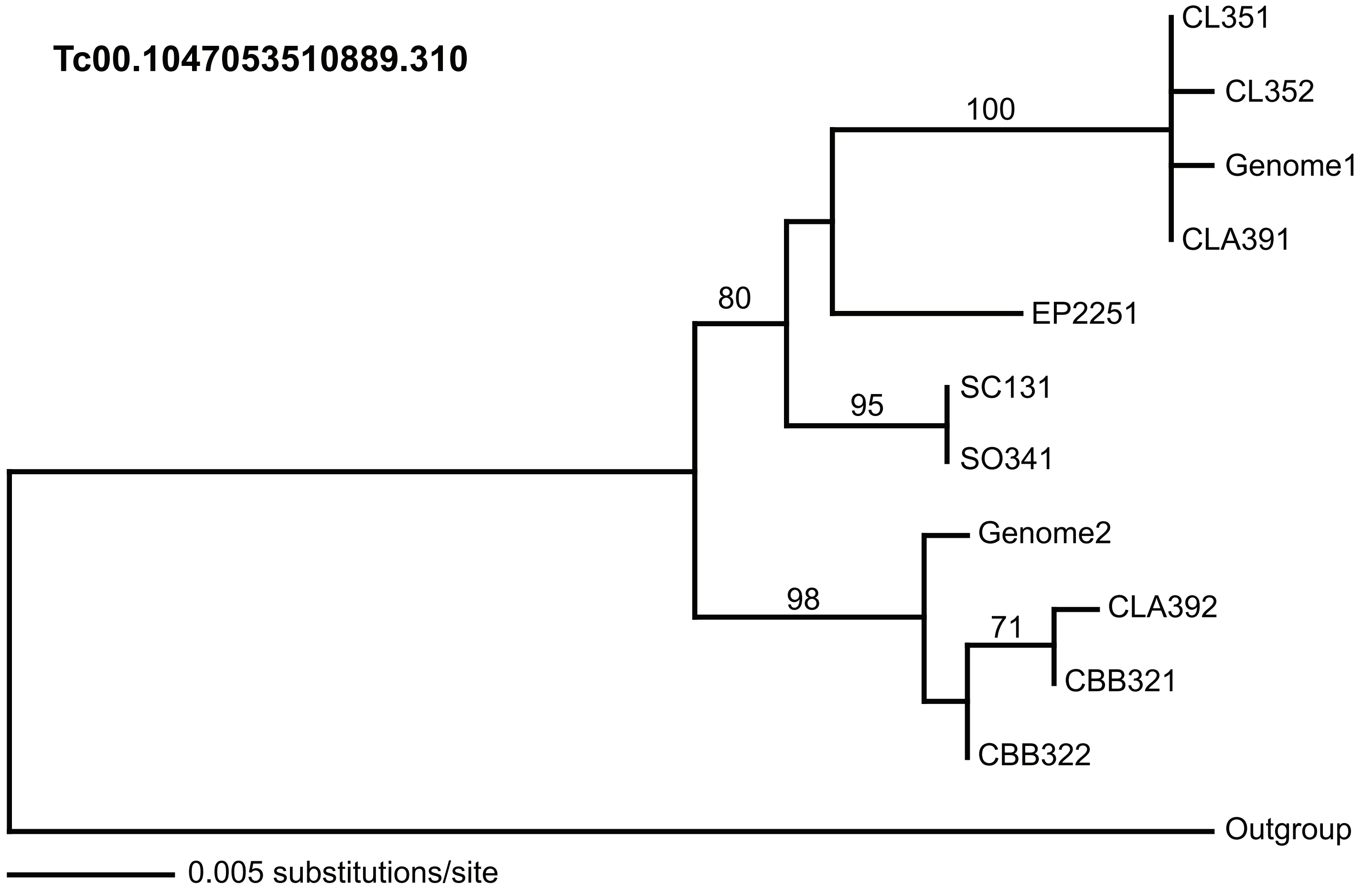
Tc00.1047053510889.310
100
80
95
98
71
CL351
CL352
Genome1
CLA391
EP2251
SC131
SO341
Genome2
CLA392
CBB321
CBB322
Outgroup
0.005 substitutions/site

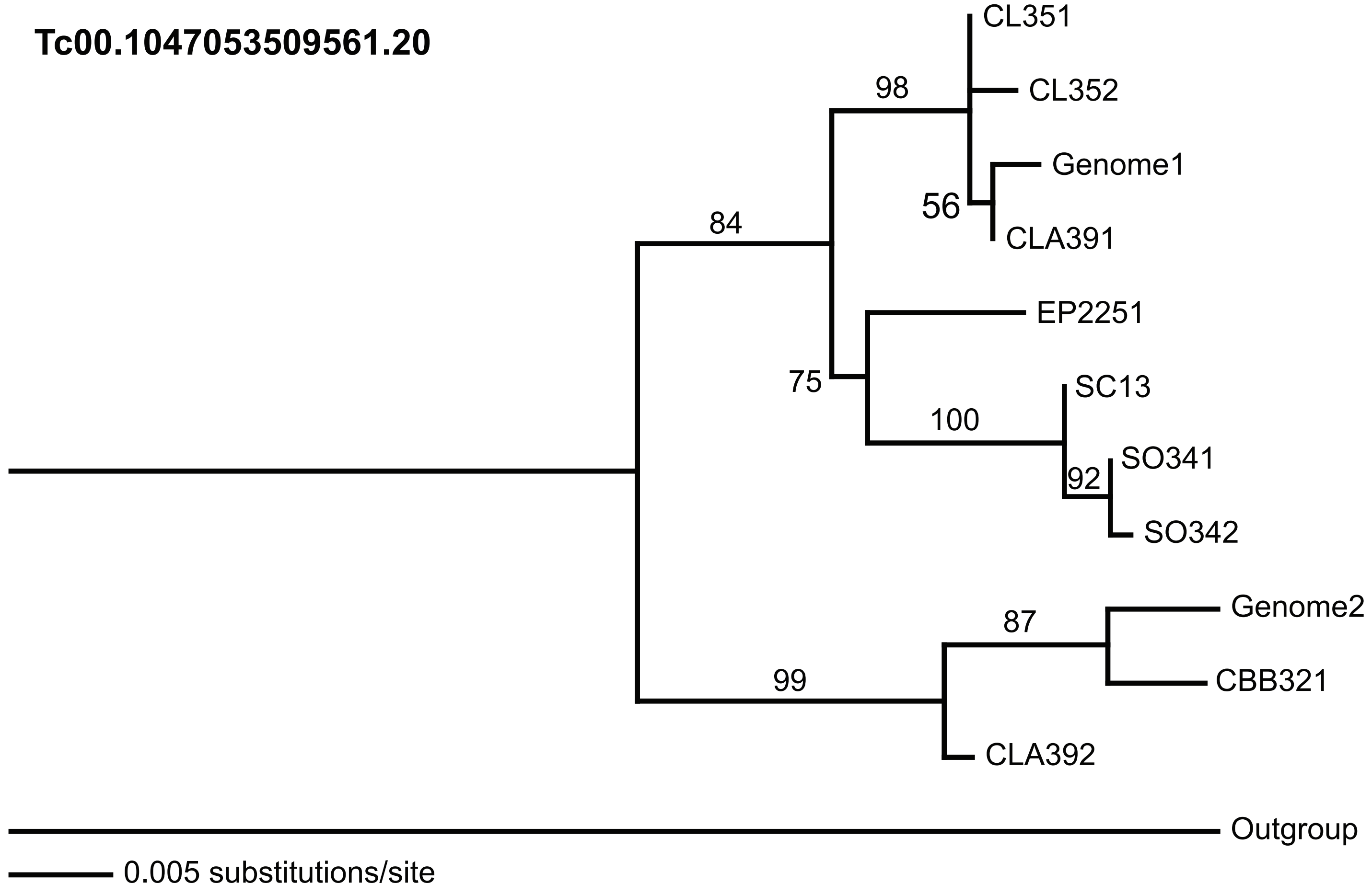
Tc00.1047053509561.20
98
56
84
75
100
92
87
99
CL351
CL352
Genome1
CLA391
EP2251
SC13
SO341
SO342
Genome2
CBB321
CLA392
Outgroup
0.005 substitutions/site

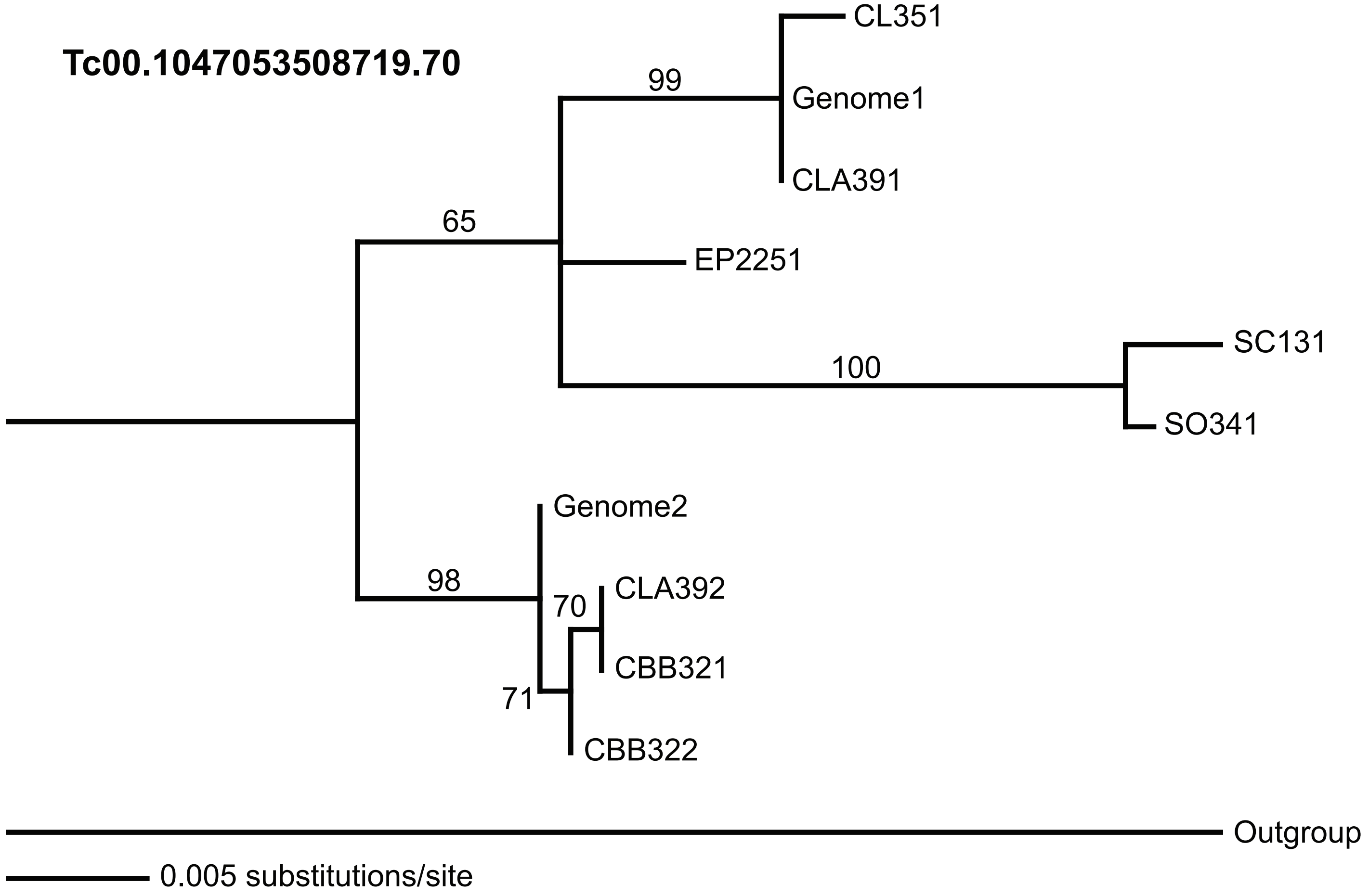
Tc00.1047053508719.70
CL351
99
Genome1
CLA391
65
EP2251
SC131
100
SO341
Genome2
98
CLA392
70
CBB321
71
CBB322
Outgroup
0.005 substitutions/site

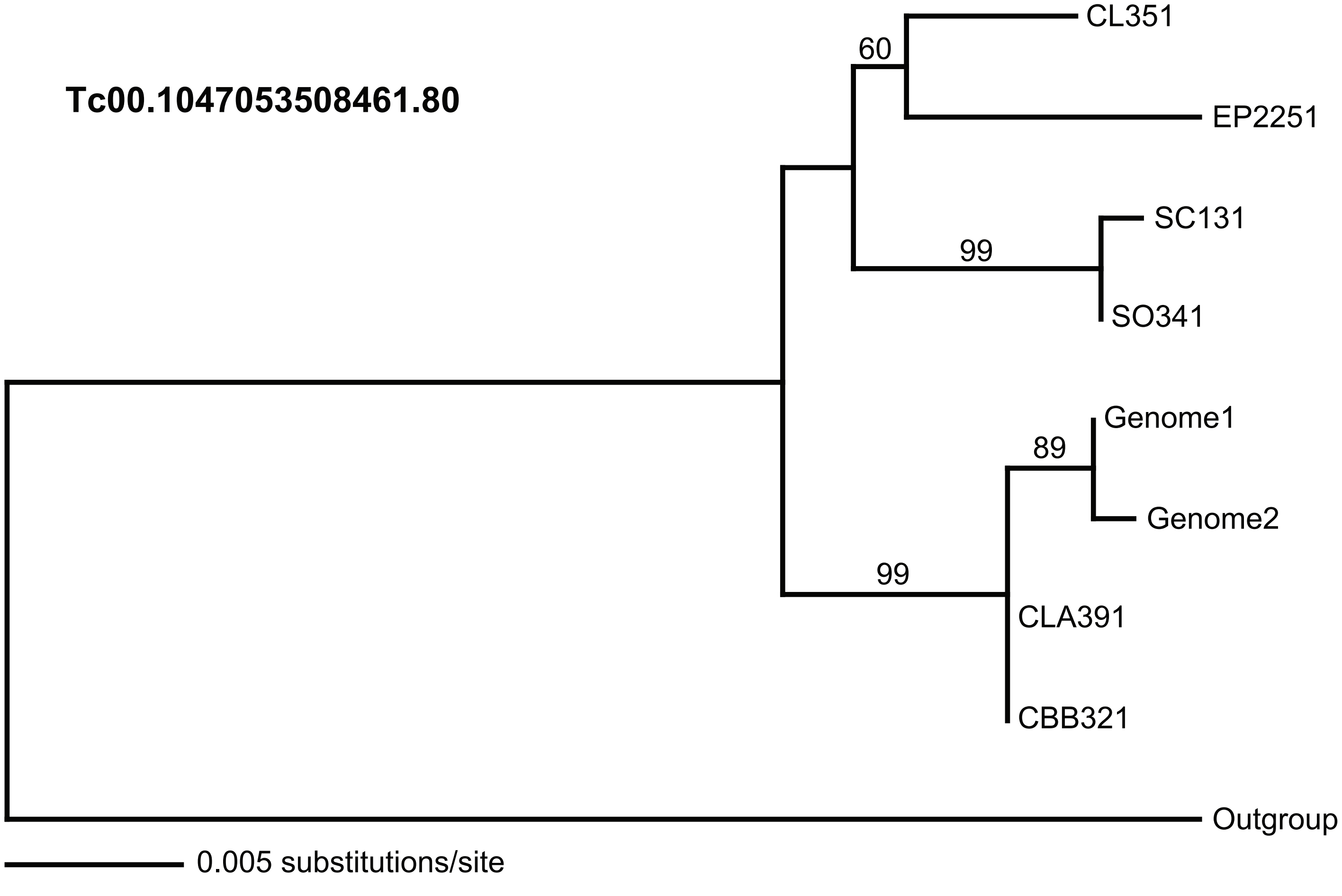
Tc00.1047053508461.80
CL351
60
EP2251
SC131
99
SO341
Genome1
89
Genome2
99
CLA391
CBB321
Outgroup
0.005 substitutions/site

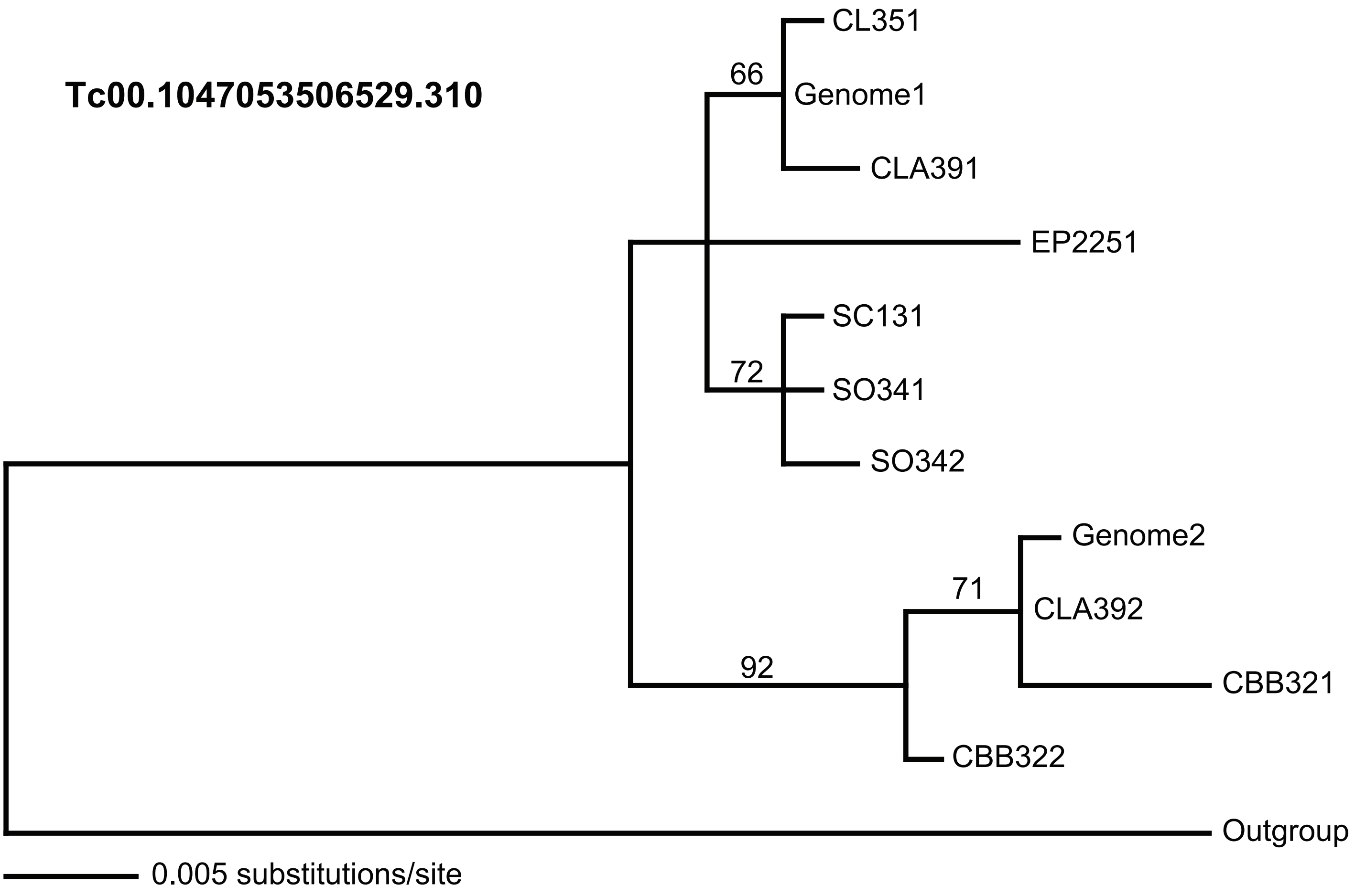
Tc00.1047053506529.310
CL351
66
Genome1
CLA391
EP2251
SC131
72
SO341
SO342
Genome2
71
CLA392
92
CBB321
CBB322
Outgroup
0.005 substitutions/site

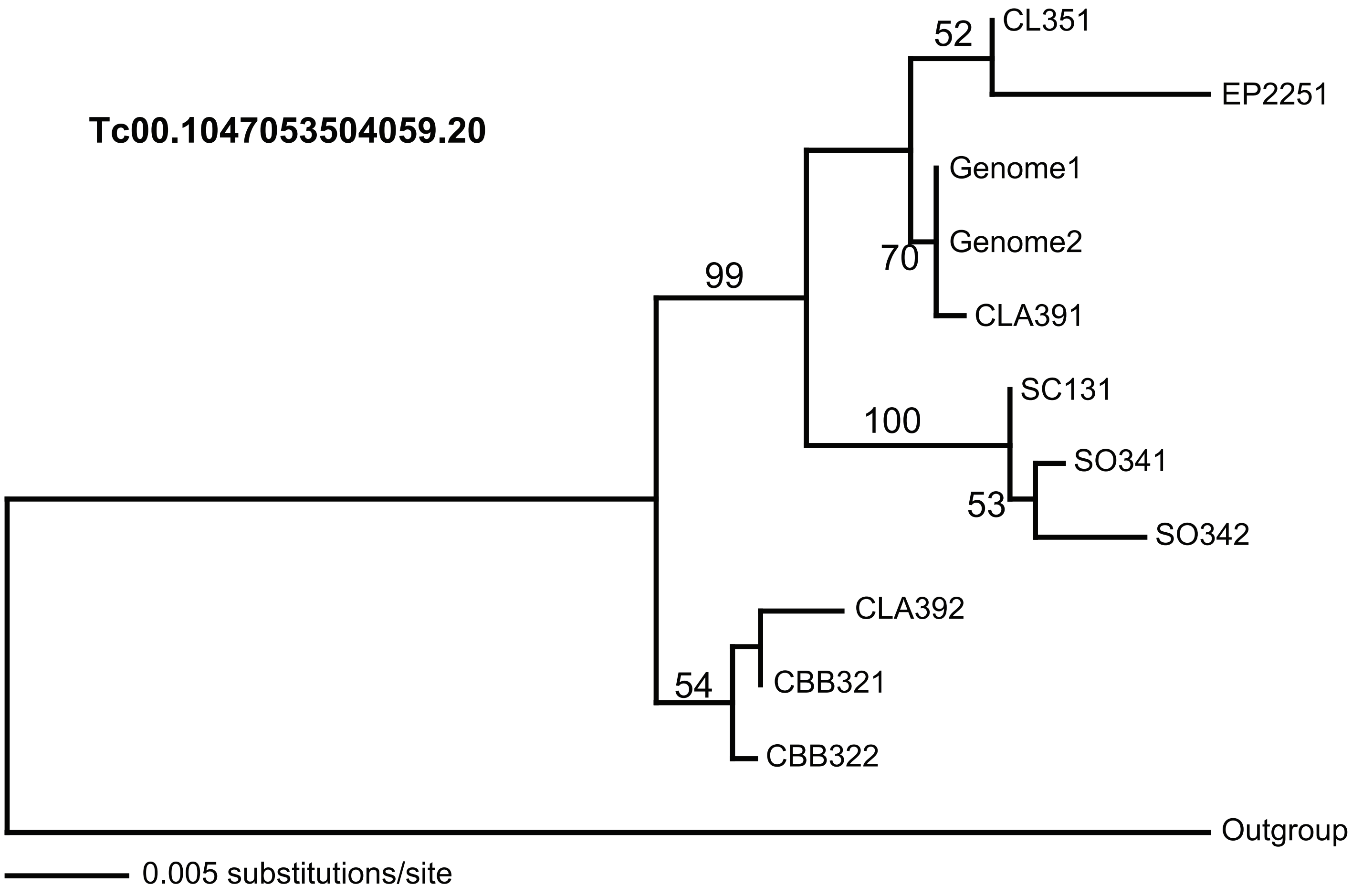
Tc00.1047053504059.20
52
CL351
EP2251
Genome1
70
Genome2
99
CLA391
SC131
100
SO341
53
SO342
CLA392
54
CBB321
CBB322
Outgroup
0.005 substitutions/site

Tc00.1047053504013.40

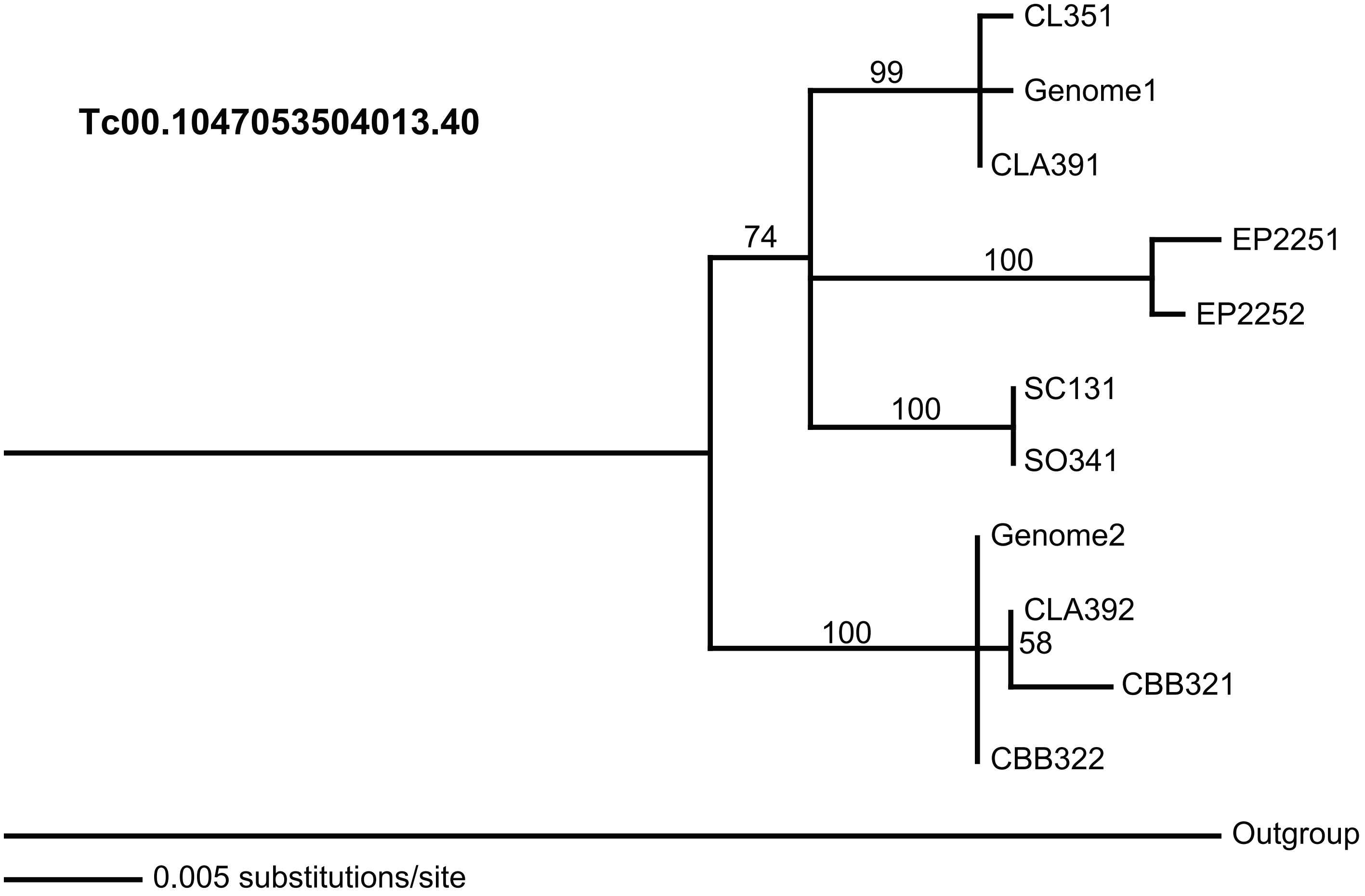

Tc00.1047053503909.76

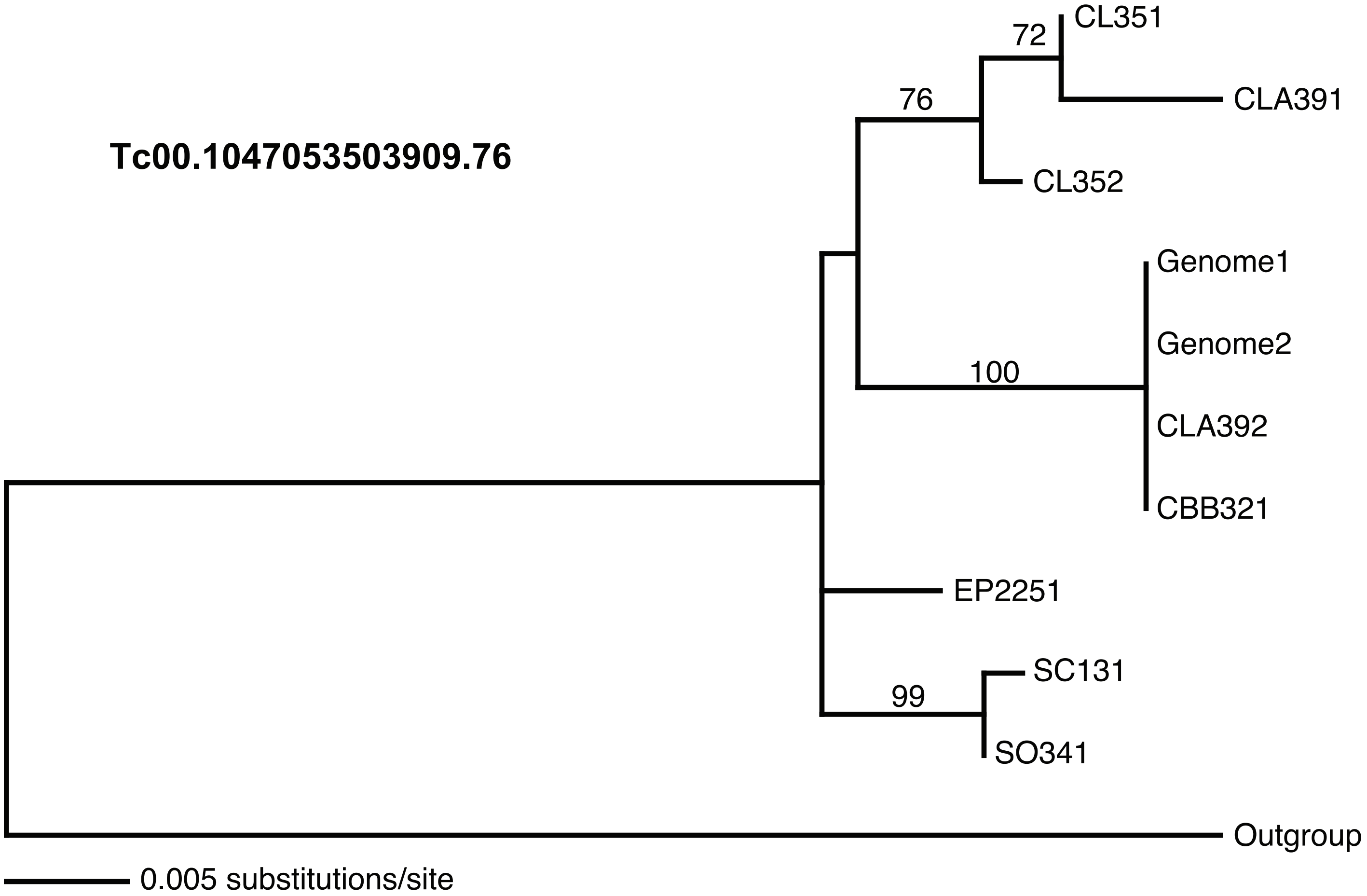

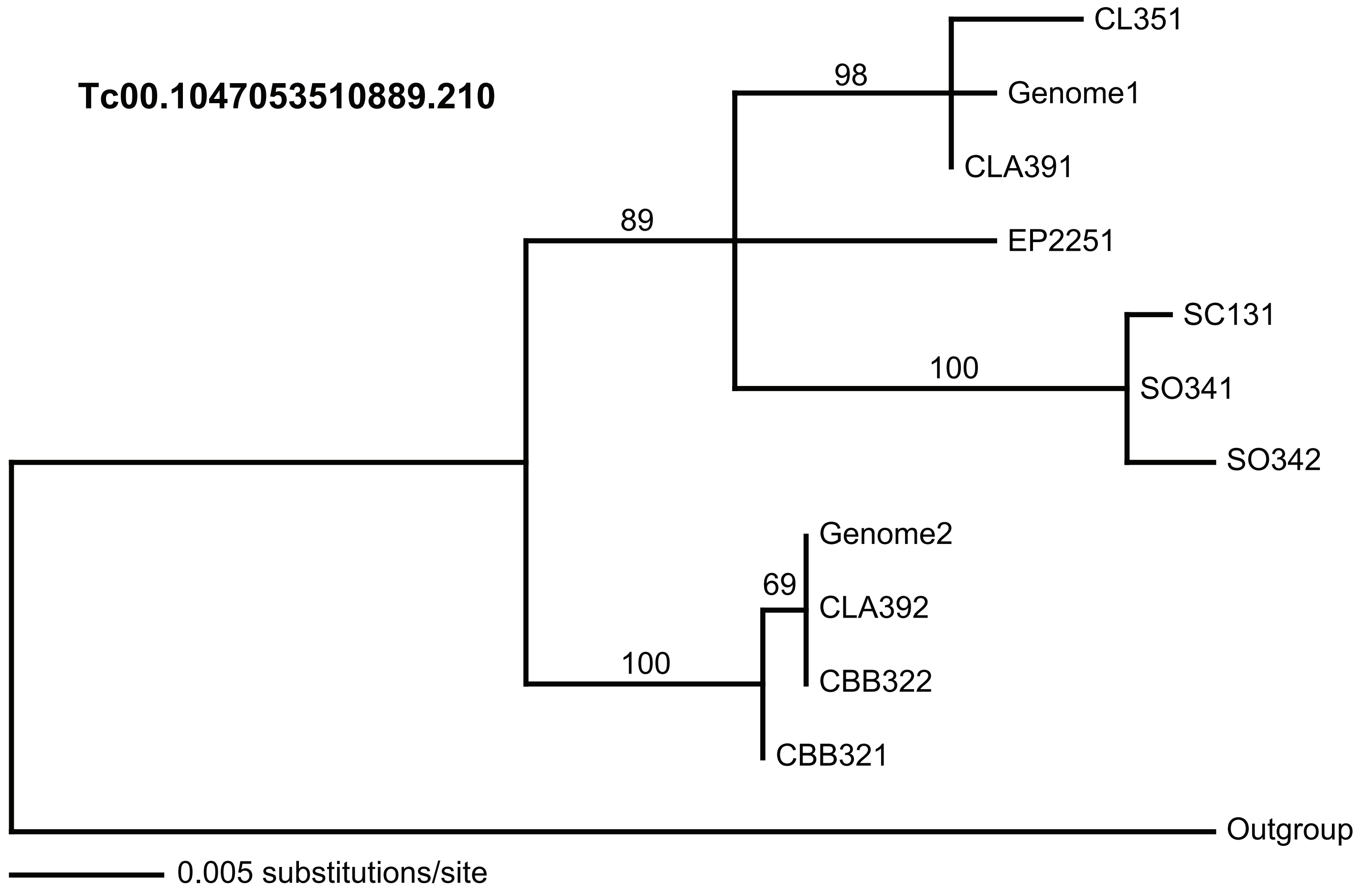

Tc00.1047053510889.210
98
89
100
69
100
CL351
Genome1
CLA391
EP2251
SC131
SO341
SO342
Genome2
CLA392
CBB322
CBB321
Outgroup
0.005 substitutions/site

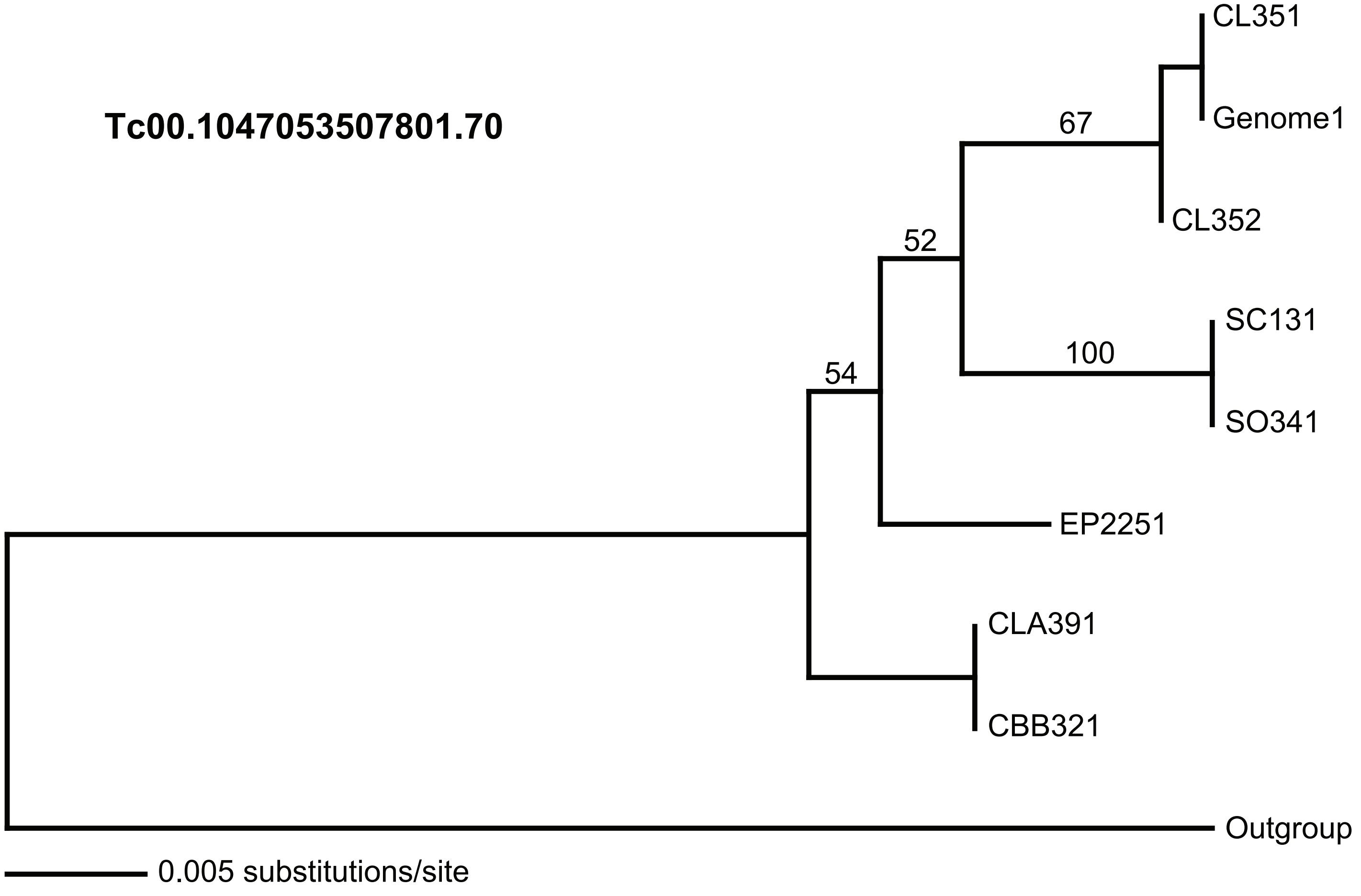

Tc00.1047053507801.70
67
52
54
100
CL351
Genome1
CL352
SC131
SO341
EP2251
CLA391
CBB321
Outgroup
0.005 substitutions/site

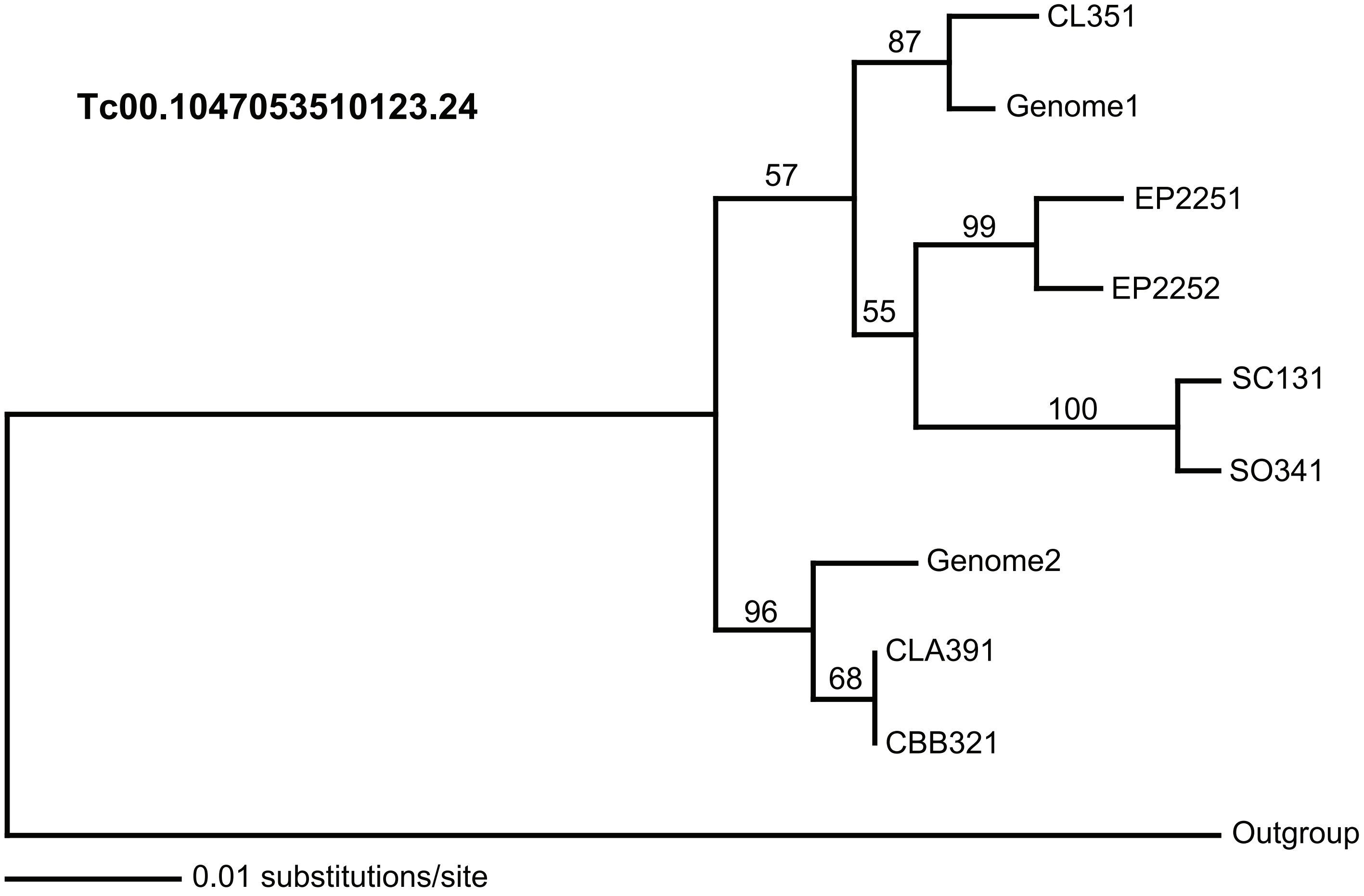
Tc00.1047053510123.24
87
57
99
55
100
96
68
CL351
Genome1
EP2251
EP2252
SC131
SO341
Genome2
CLA391
CBB321
Outgroup
0.01 substitutions/site

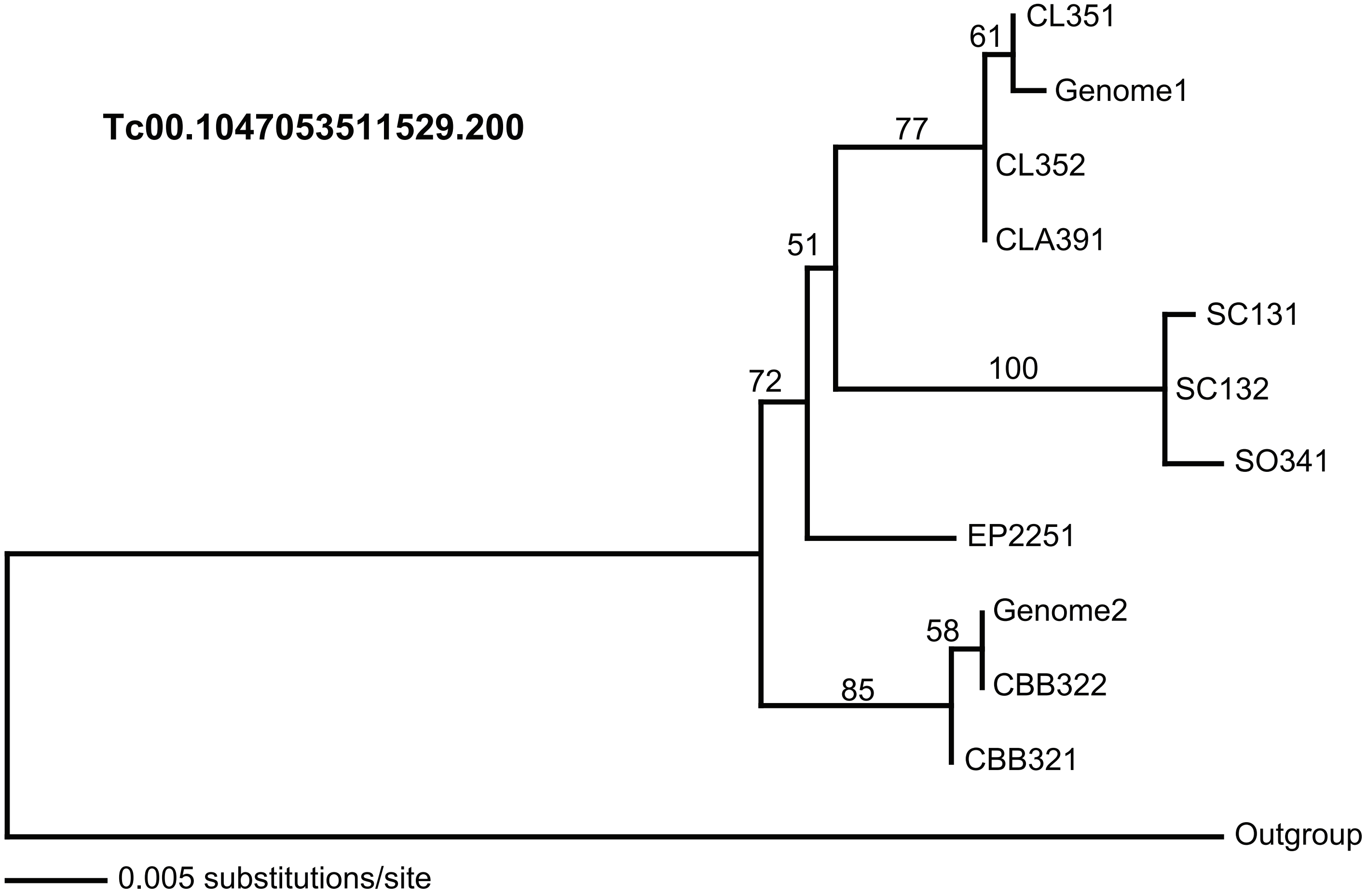
Tc00.1047053511529.200
61
77
51
72
100
58
85
CL351
Genome1
CL352
CLA391
SC131
SC132
SO341
EP2251
Genome2
CBB322
CBB321
Outgroup
0.005 substitutions/site

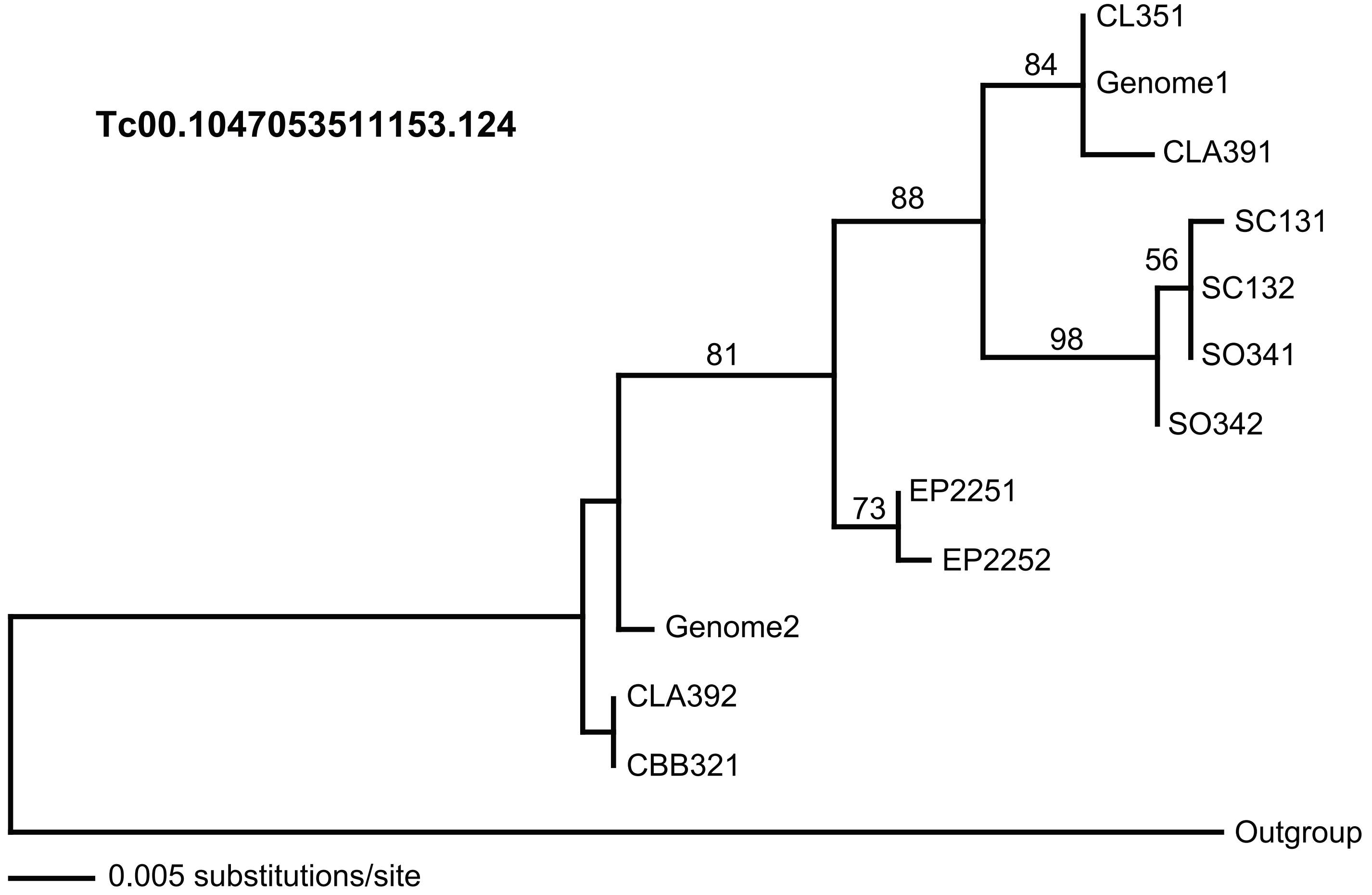
Tc00.1047053511153.124
CL351
Genome1
CLA391
84
88
SC131
56
SC132
SO341
98
SO342
81
EP2251
73
EP2252
Genome2
CLA392
CBB321
Outgroup
0.005 substitutions/site

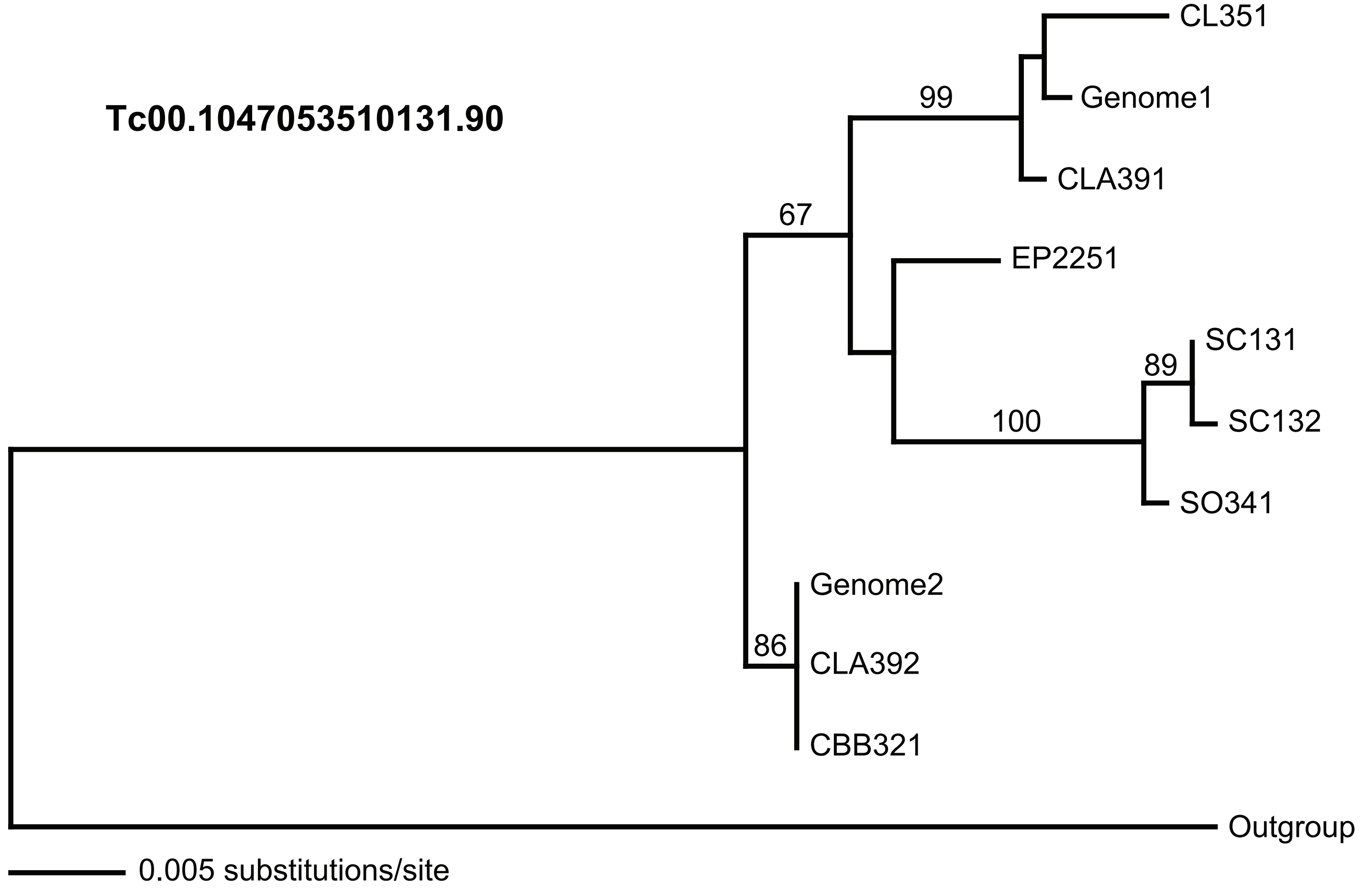
Tc00.1047053510131.90
CL351
Genome1
99
CLA391
67
EP2251
SC131
89
SC132
100
SO341
Genome2
86
CLA392
CBB321
Outgroup
0.005 substitutions/site

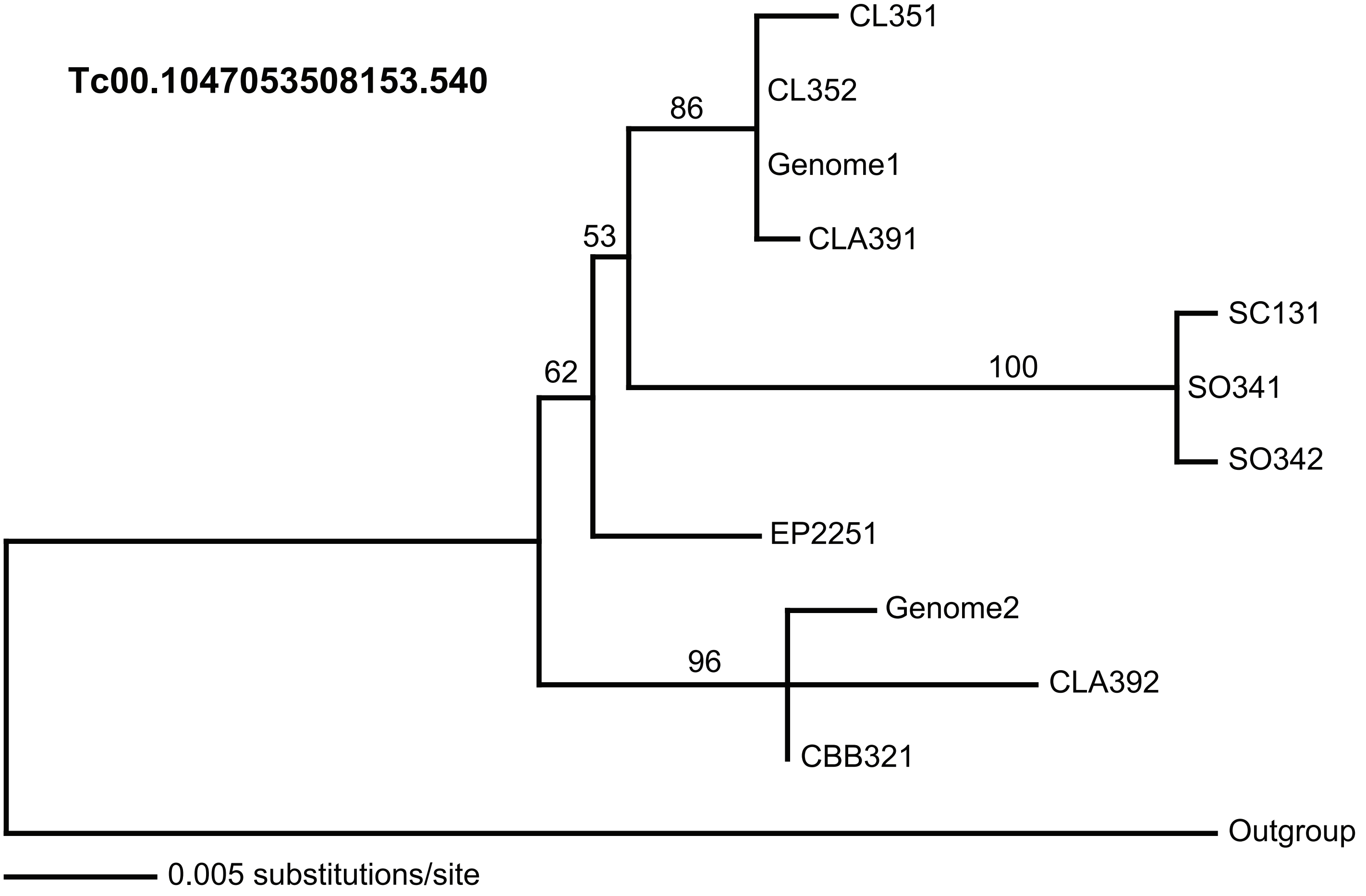
Tc00.1047053508153.540
CL351
CL352
Genome1
CLA391
86
53
SC131
SO341
SO342
100
62
EP2251
Genome2
96
CLA392
CBB321
Outgroup
0.005 substitutions/site

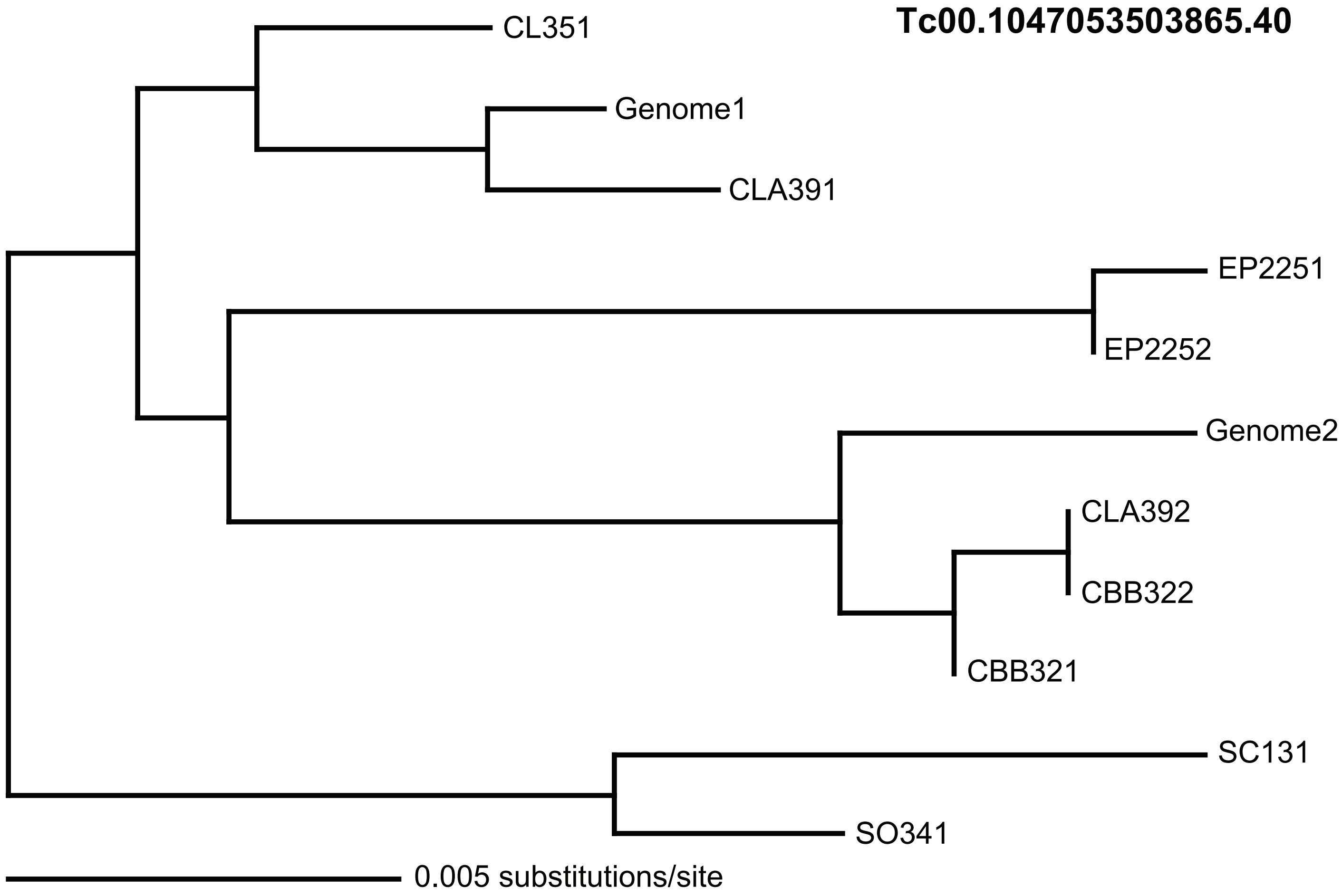
Tc00.1047053503865.40
CL351
Genome1
CLA391
EP2251
EP2252
Genome2
CLA392
CBB322
CBB321
SC131
SO341
0.005 substitutions/site

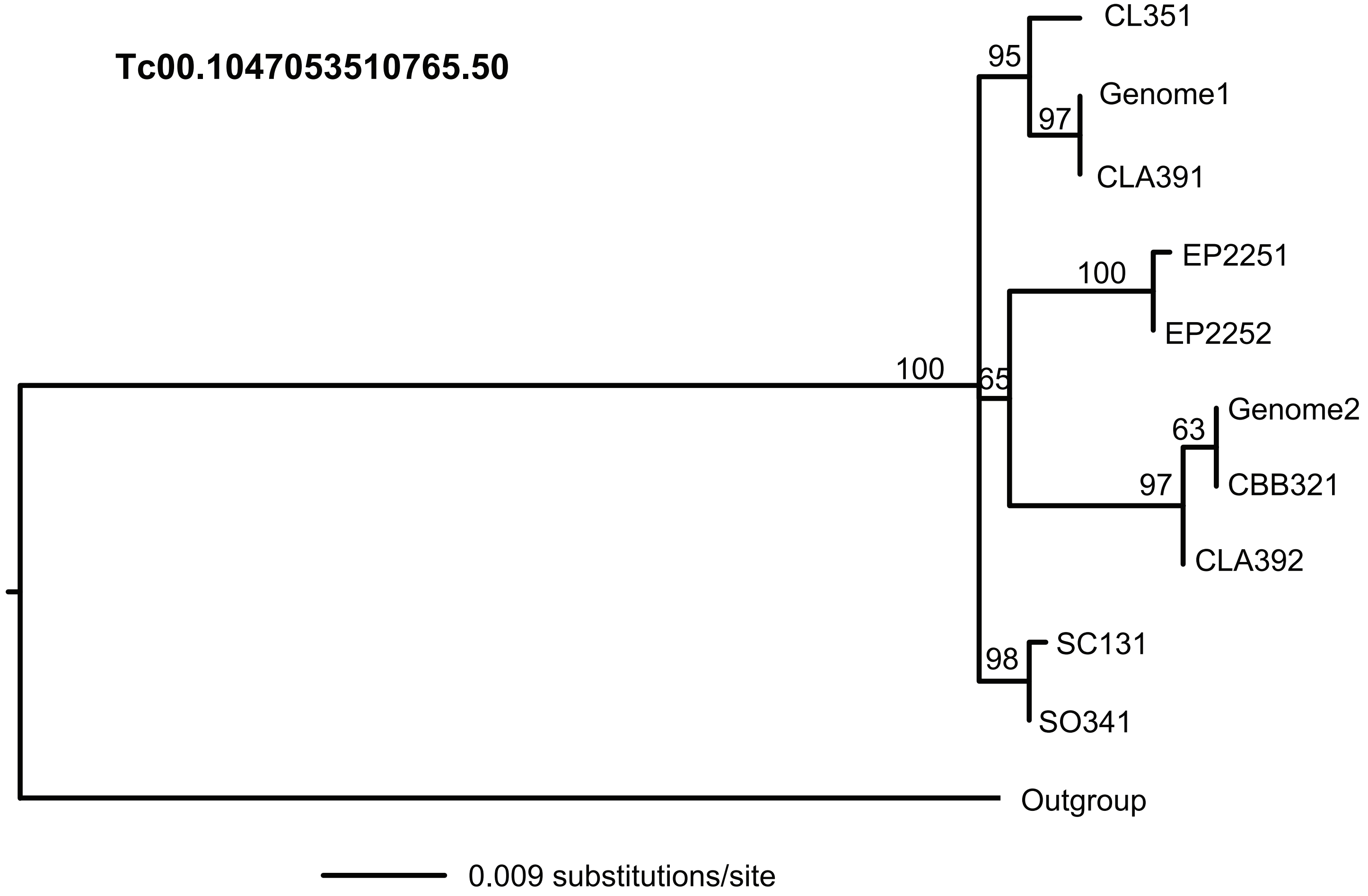
Tc00.1047053510765.50
CL351
Genome1
CLA391
EP2251
EP2252
Genome2
CBB321
CLA392
SC131
SO341
Outgroup
95
97
100
100
65
63
97
98
0.009 substitutions/site

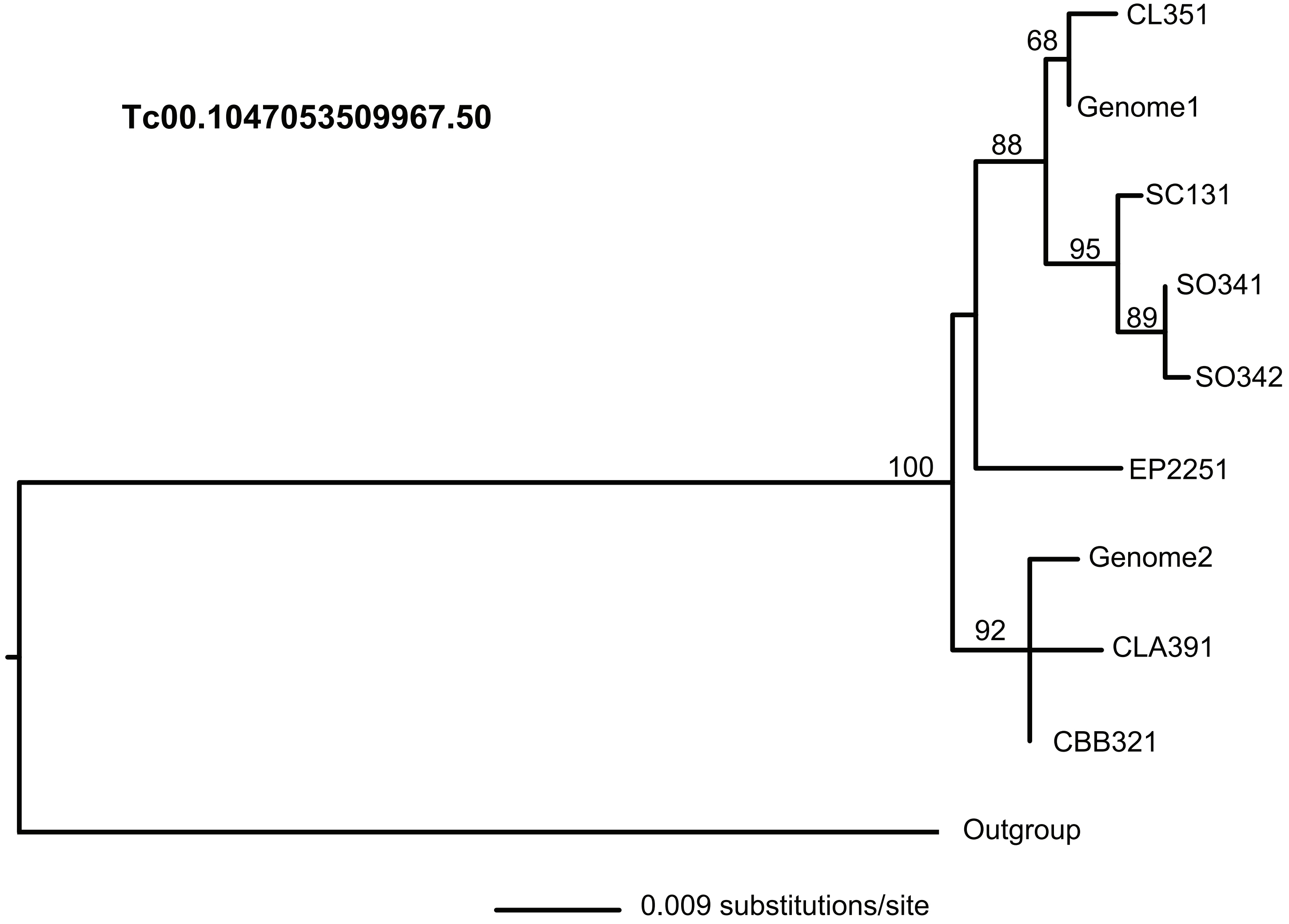
Tc00.1047053509967.50
CL351
Genome1
SC131
SO341
SO342
EP2251
Genome2
CLA391
CBB321
Outgroup
68
88
95
89
100
92
0.009 substitutions/site

Tc00.1047053510101.480

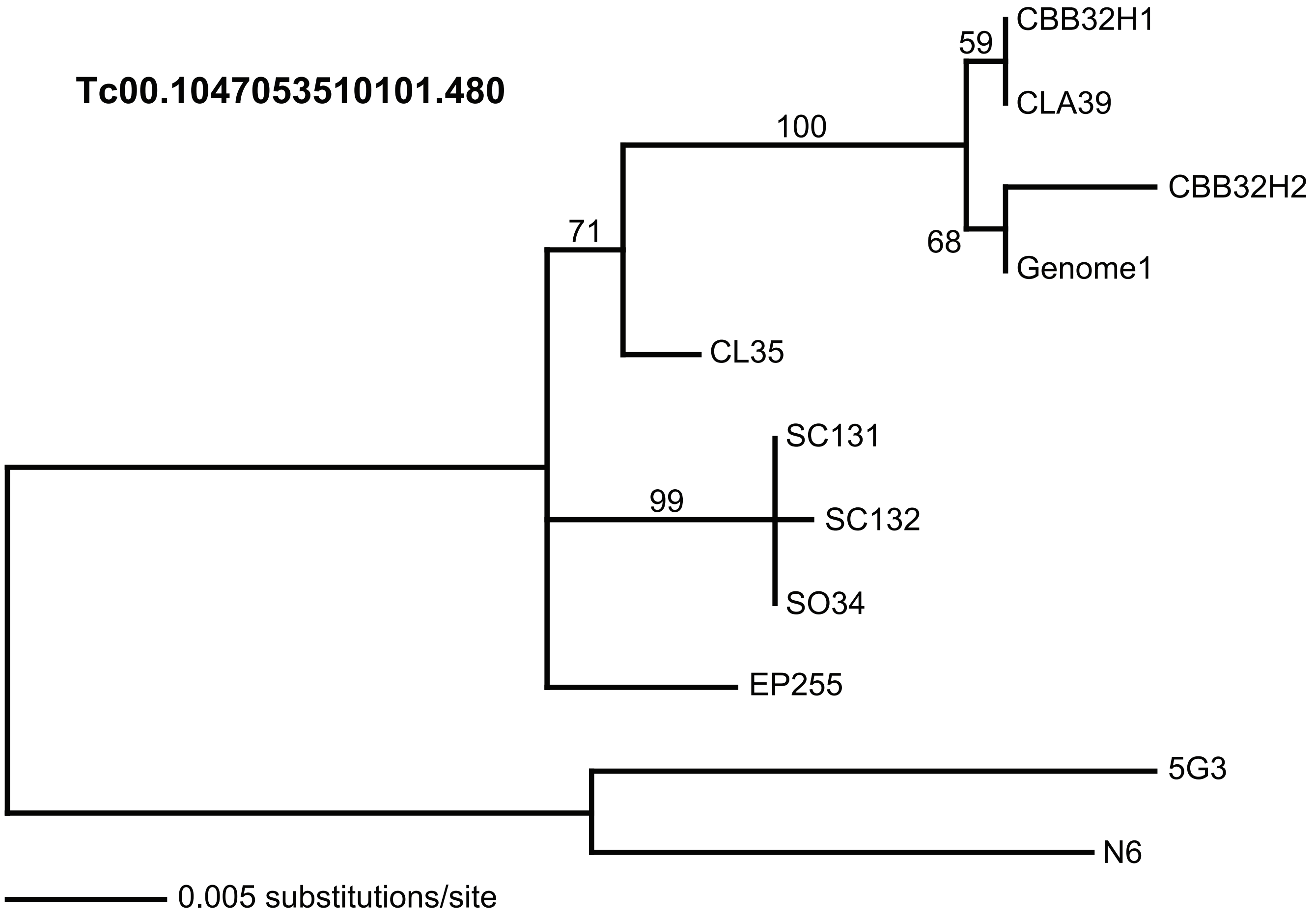

Tc00.1047053506525.150

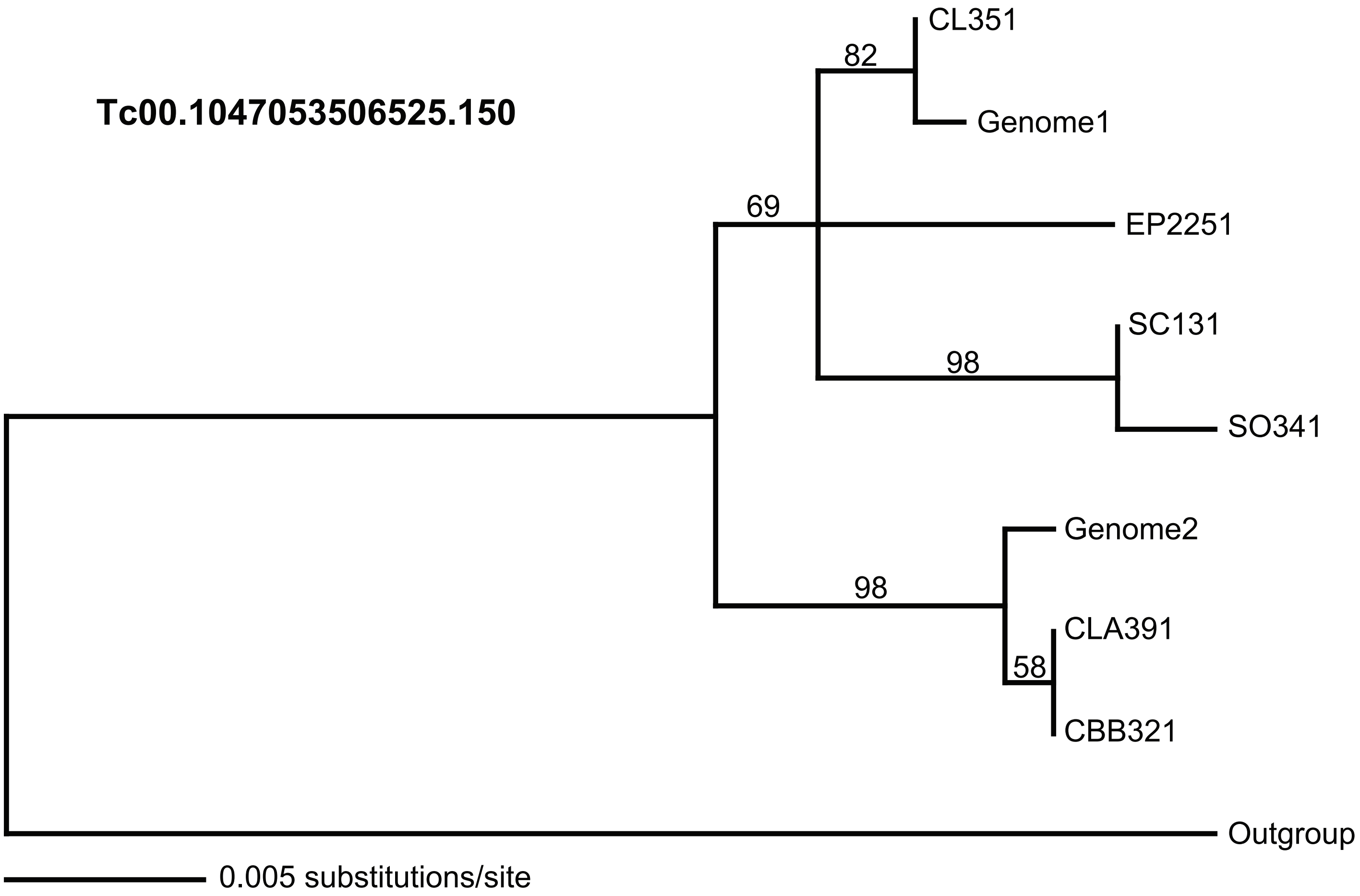

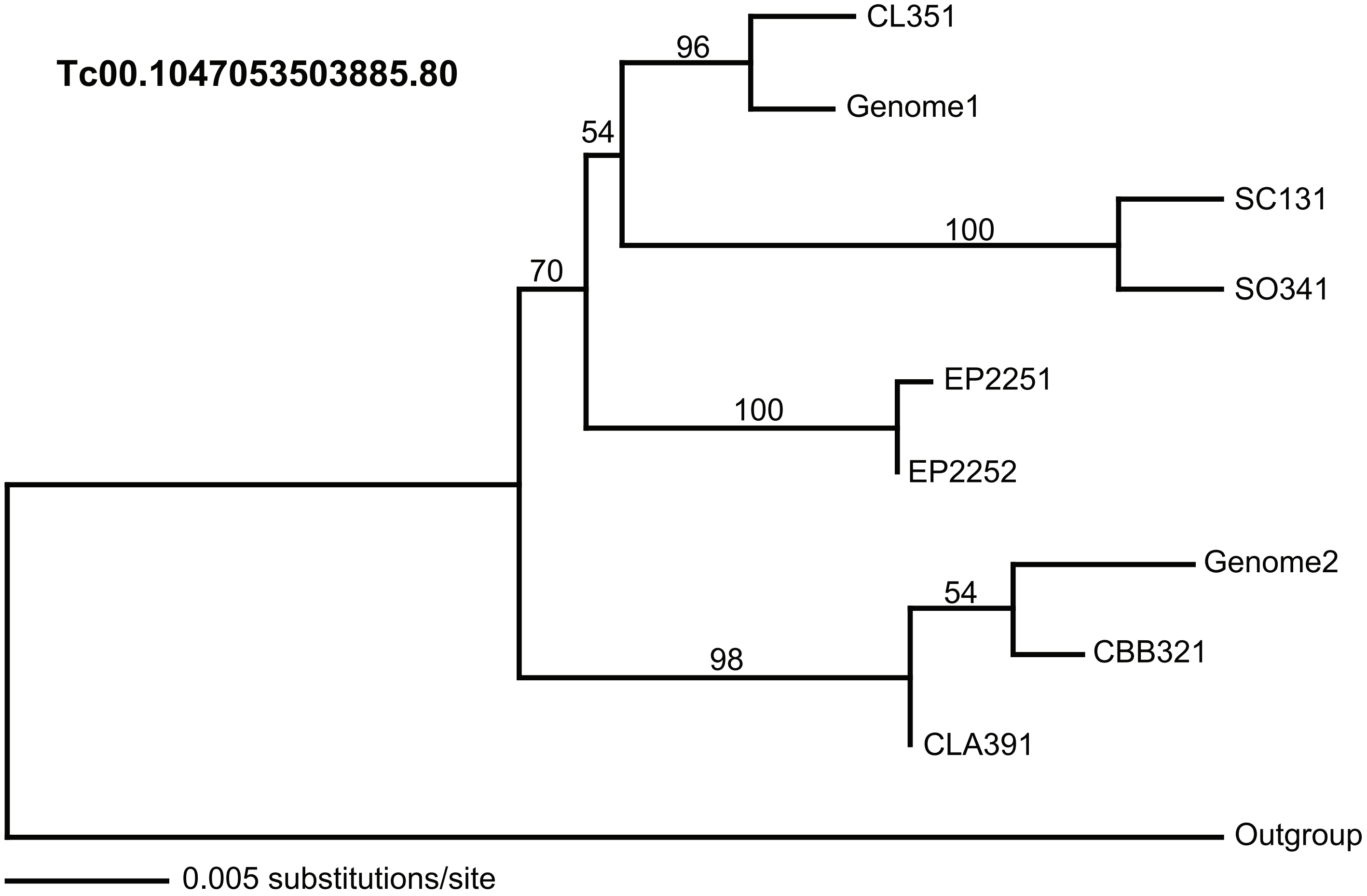
Tc00.1047053503885.80
CL351
Genome1
SC131
SO341
EP2251
EP2252
Genome2
CBB321
CLA391
Outgroup
96
54
70
100
100
54
98
0.005 substitutions/site

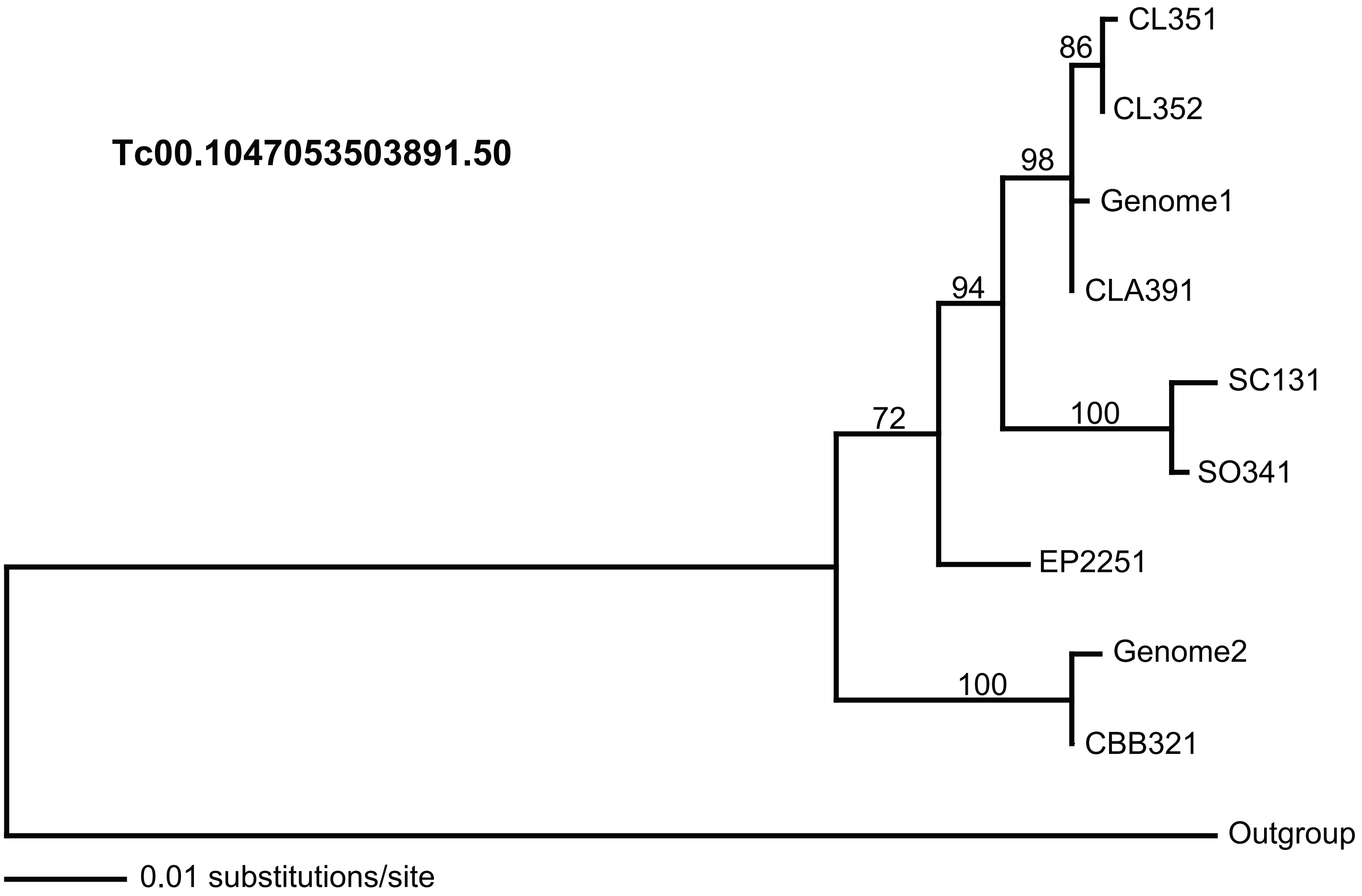
Tc00.1047053503891.50
CL351
CL352
Genome1
CLA391
SC131
SO341
EP2251
Genome2
CBB321
Outgroup
86
98
94
72
100
100
0.01 substitutions/site

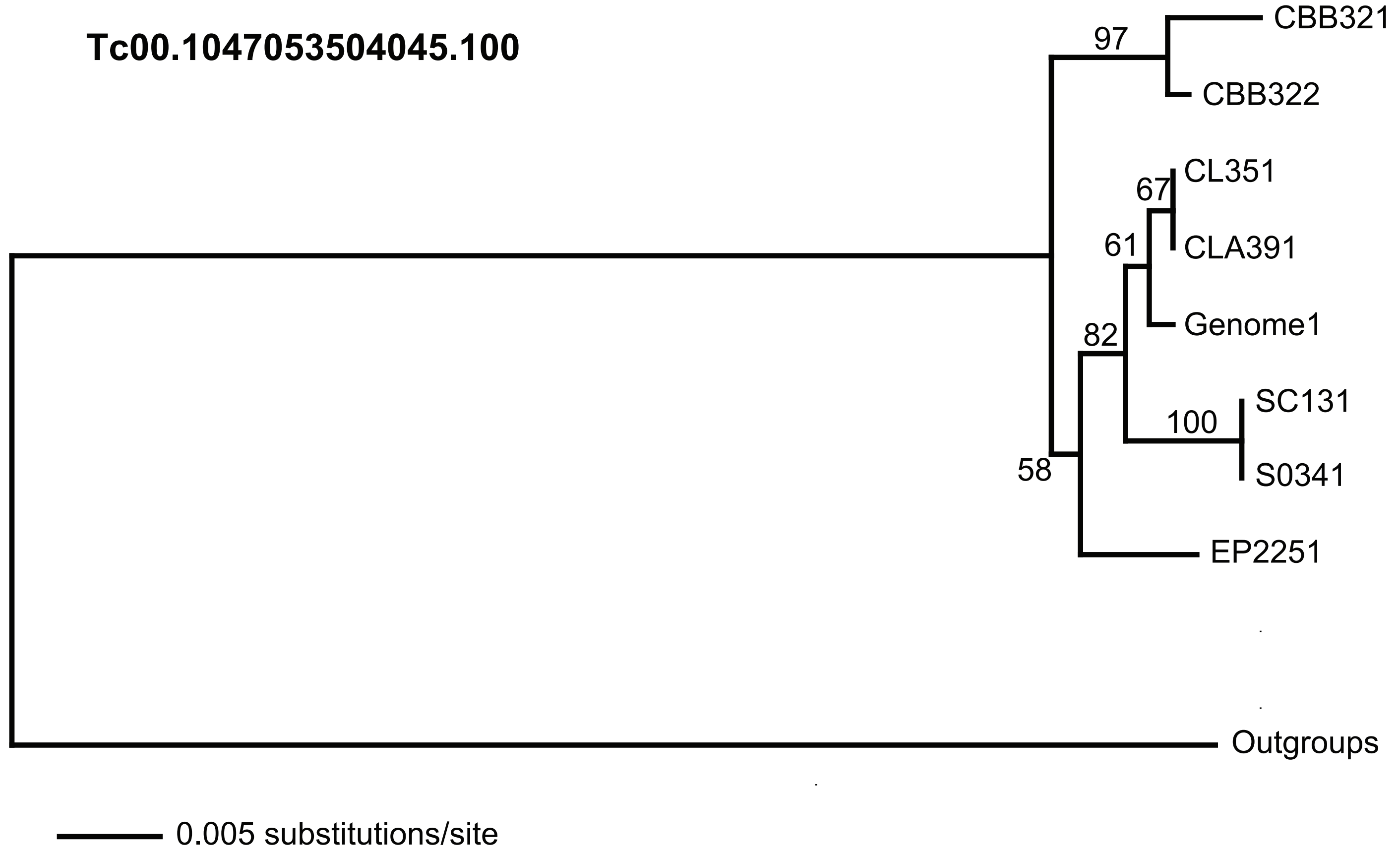
Tc00.1047053504045.100
97
CBB321
CBB322
67
CL351
61
CLA391
Genome1
82
SC131
100
58
S0341
EP2251
Outgroups
0.005 substitutions/site

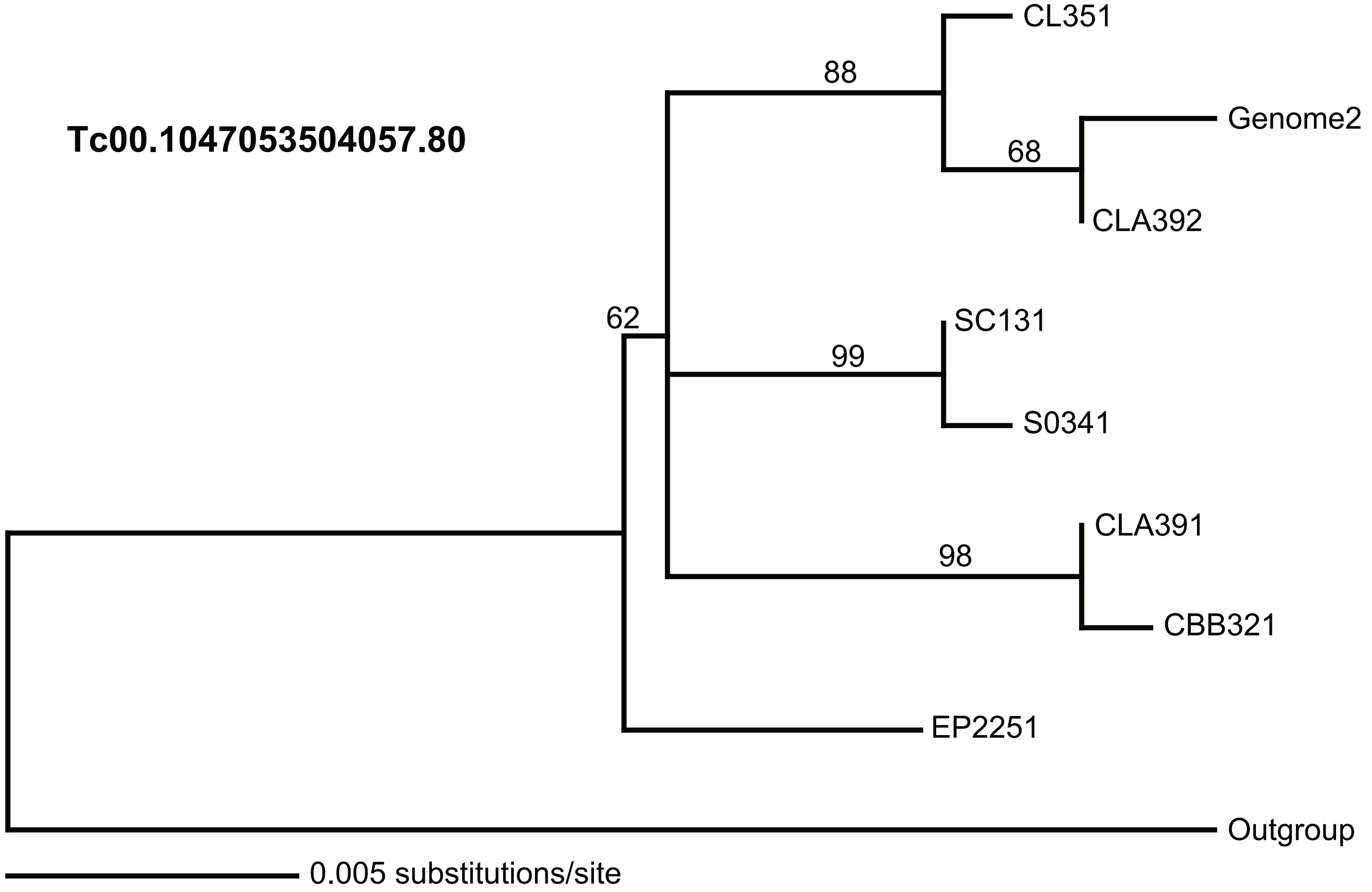
Tc00.1047053504057.80
CL351
88
Genome2
68
CLA392
62
SC131
99
S0341
CLA391
98
CBB321
EP2251
Outgroup
0.005 substitutions/site

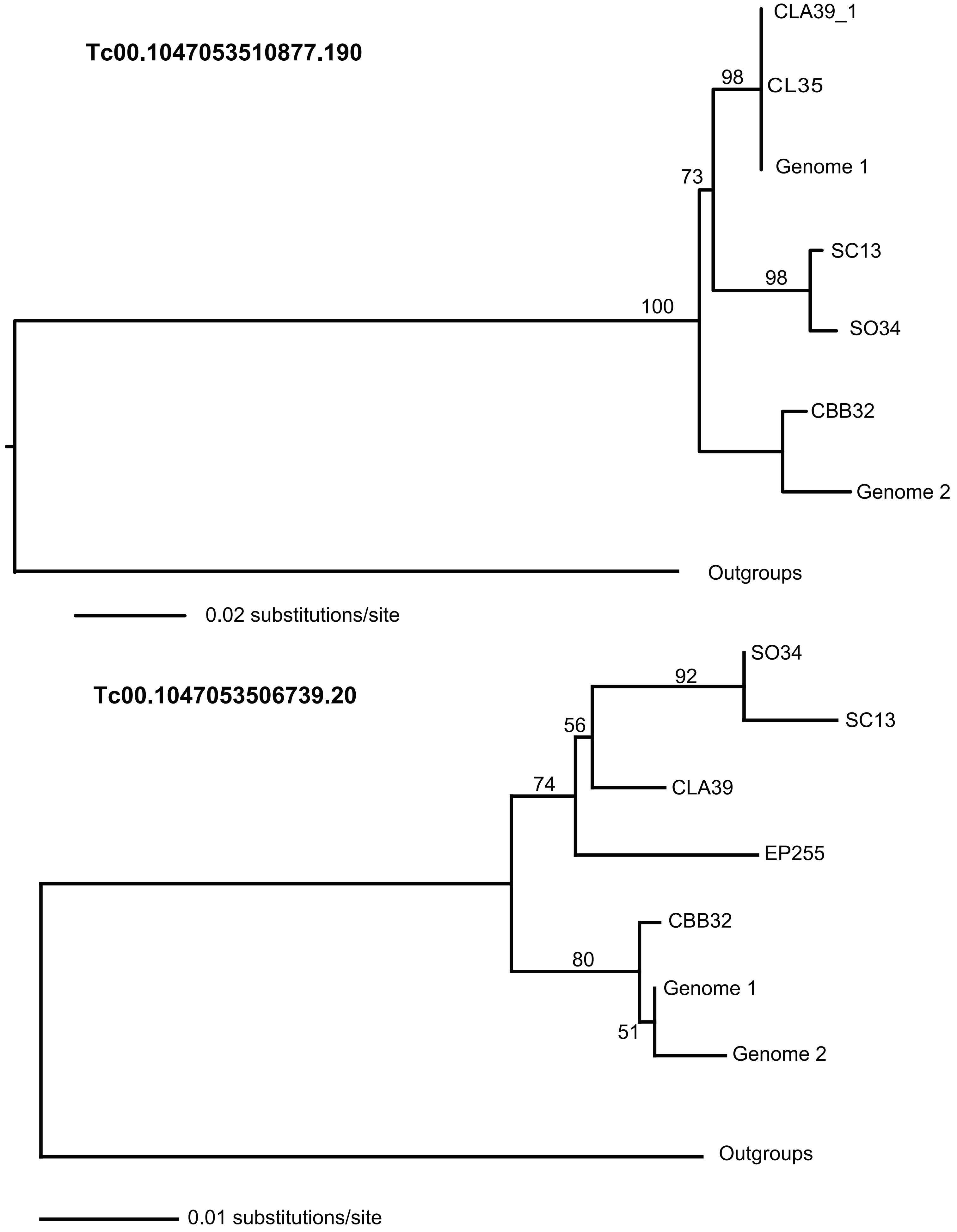
Tc00.1047053510877.190
CLA39_1
98
CL35
Genome 1
73
SC13
98
SO34
100
CBB32
Genome 2
Outgroups
0.02 substitutions/site
Tc00.1047053506739.20
SO34
92
SC13
56
CLA39
74
EP255
CBB32
80
Genome 1
51
Genome 2
Outgroups
0.01 substitutions/site

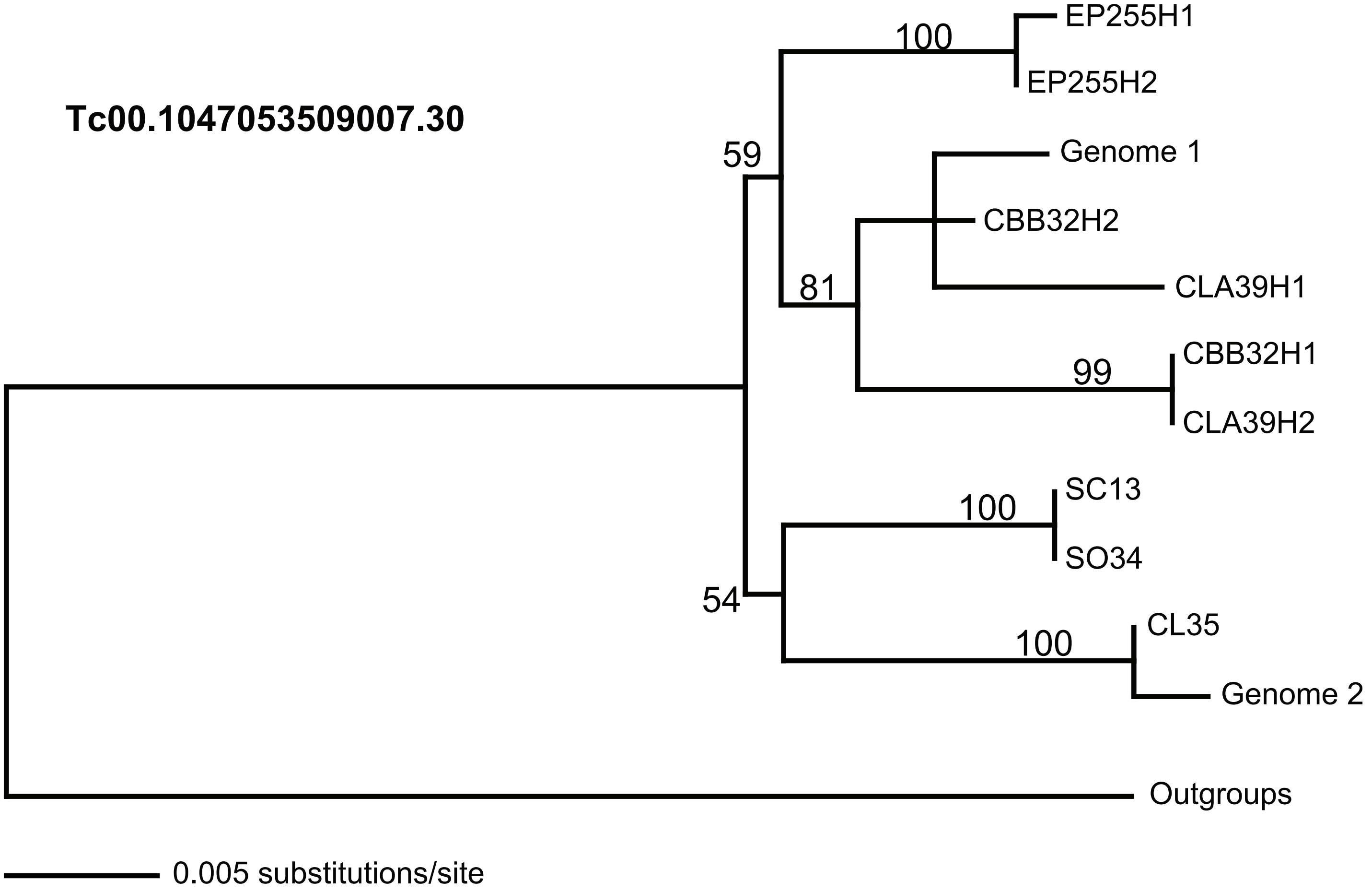
Tc00.1047053509007.30
100
EP255H1
EP255H2
59
Genome 1
CBB32H2
81
CLA39H1
99
CBB32H1
CLA39H2
100
SC13
SO34
54
100
CL35
Genome 2
Outgroups
0.005 substitutions/site

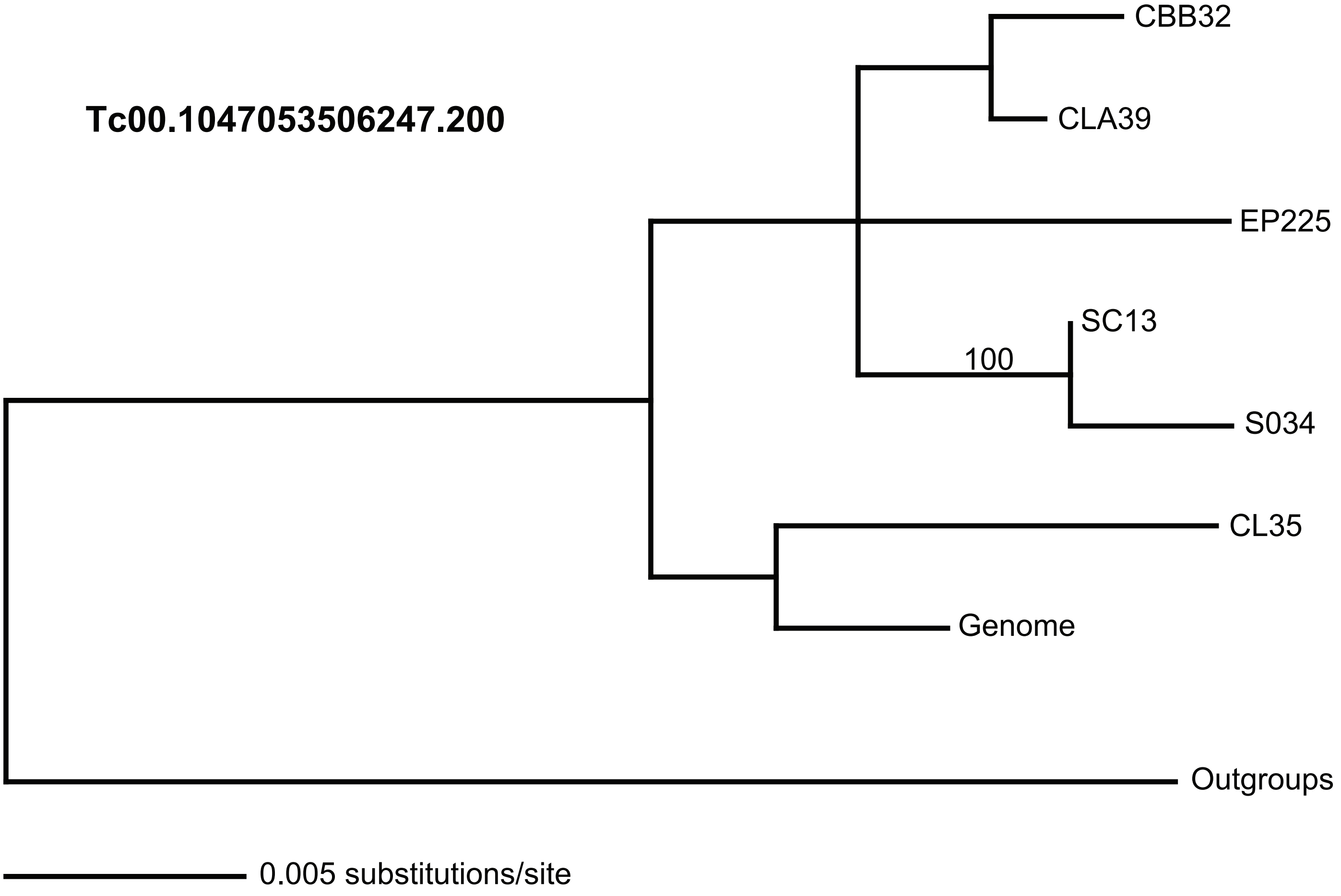
Tc00.1047053506247.200
CBB32
CLA39
EP225
100
SC13
S034
CL35
Genome
Outgroups
0.005 substitutions/site

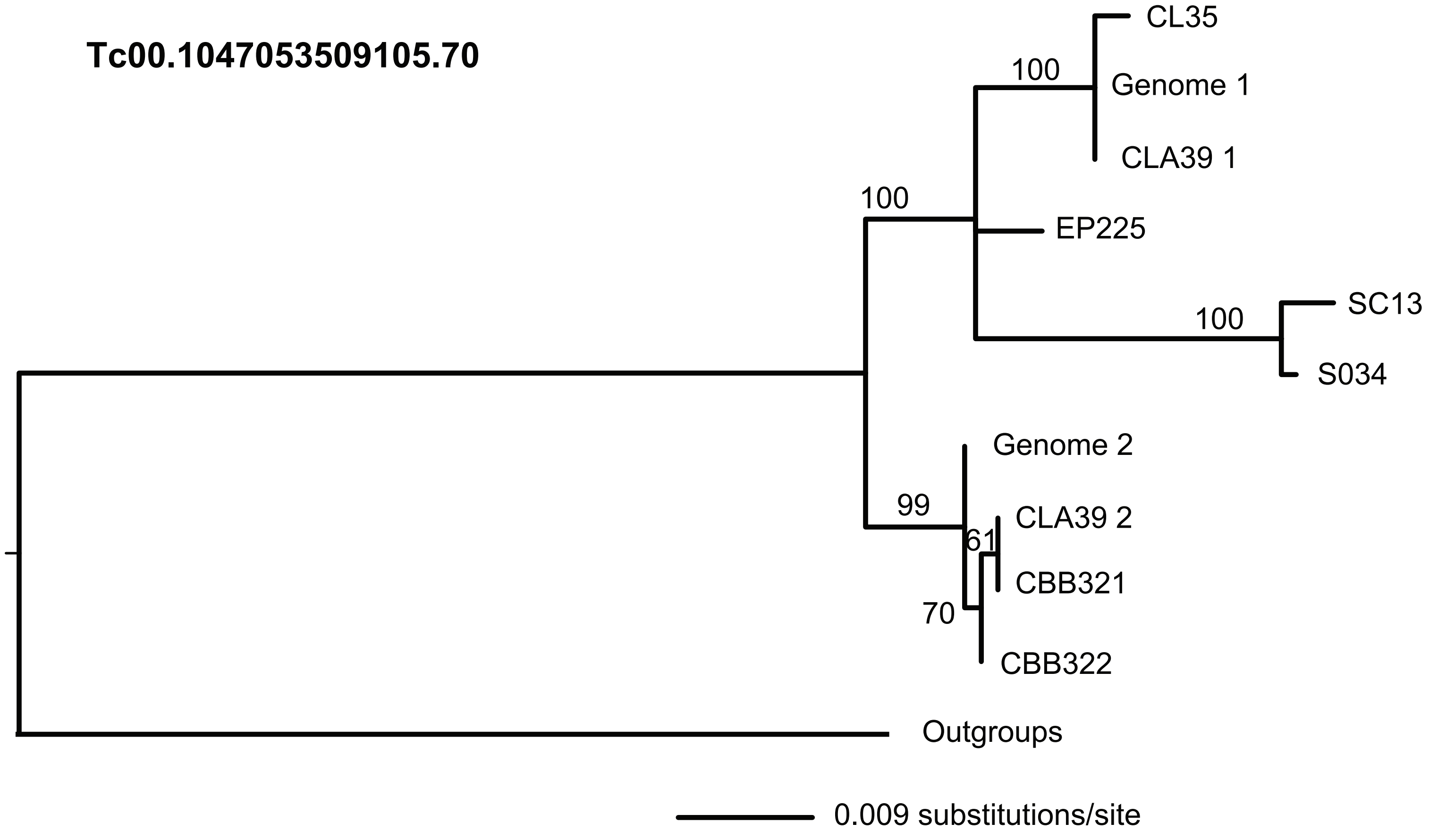
Tc00.1047053509105.70
CL35
Genome 1
CLA39 1
EP225
SC13
S034
Genome 2
CLA39 2
CBB321
CBB322
Outgroups
100
100
100
99
61
70
0.009 substitutions/site

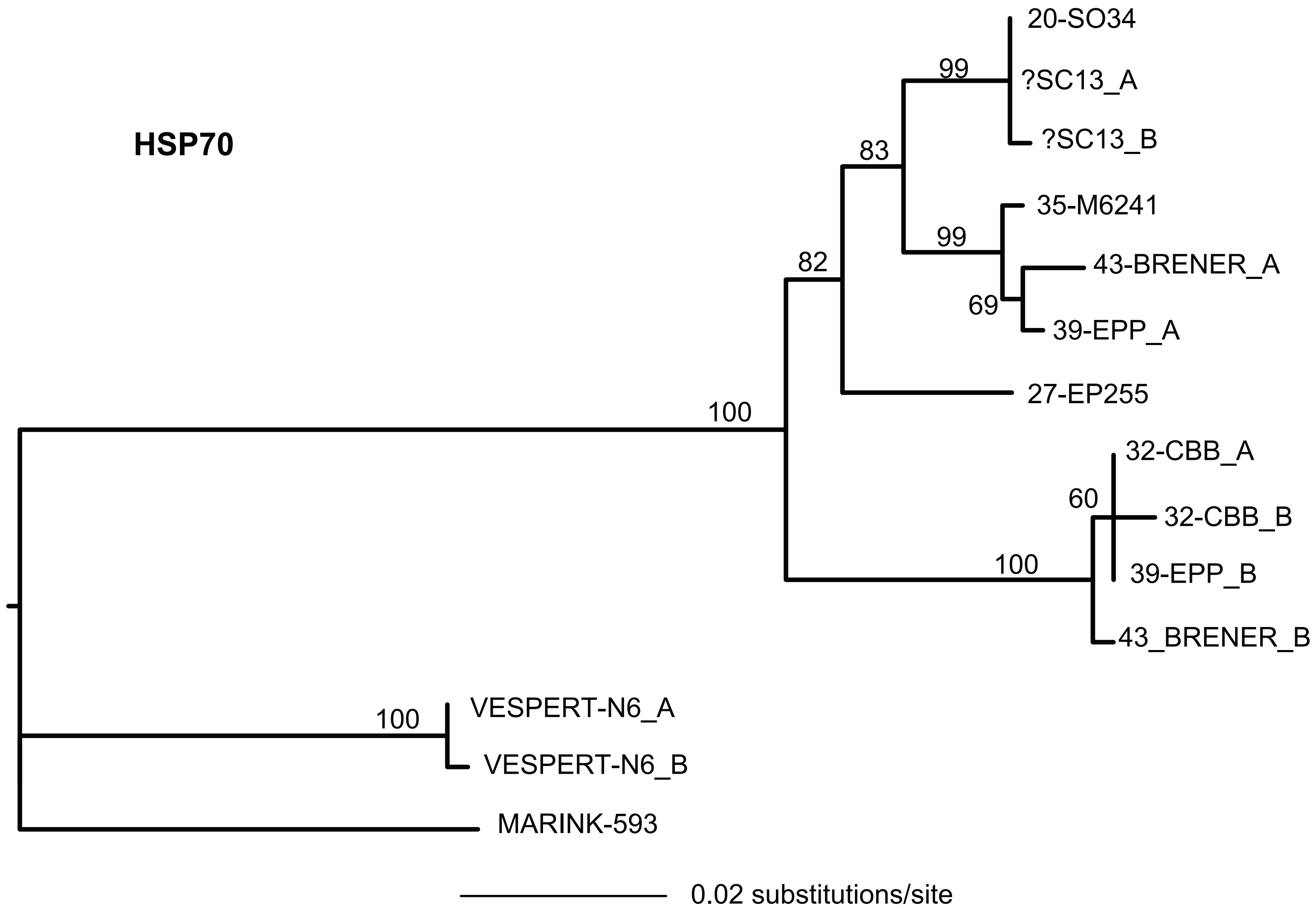
HSP70
20-SO34
?SC13_A
?SC13_B
35-M6241
43-BRENER_A
39-EPP_A
27-EP255
32-CBB_A
32-CBB_B
39-EPP_B
43_BRENER_B
VESPERT-N6_A
VESPERT-N6_B
MARINK-593
99
83
99
82
69
100
60
100
100
0.02 substitutions/site
