# Supplementary figures and images for: Analyses of 32 Loci Clarify Phylogenetic Relationships among Trypanosoma cruzi Lineages and Support a Single Hybridization prior to Human Contact
Source: PLoS Negl Trop Dis. 2011 Aug 2;5(8):e1272. doi: 10.1371/journal.pntd.0001272 (PMC3149036; doi:10.1371/journal.pntd.0001272)

Estimated Divergence times for main DTU clades of  
*T. cruzi* using nuclear loci with strict clock

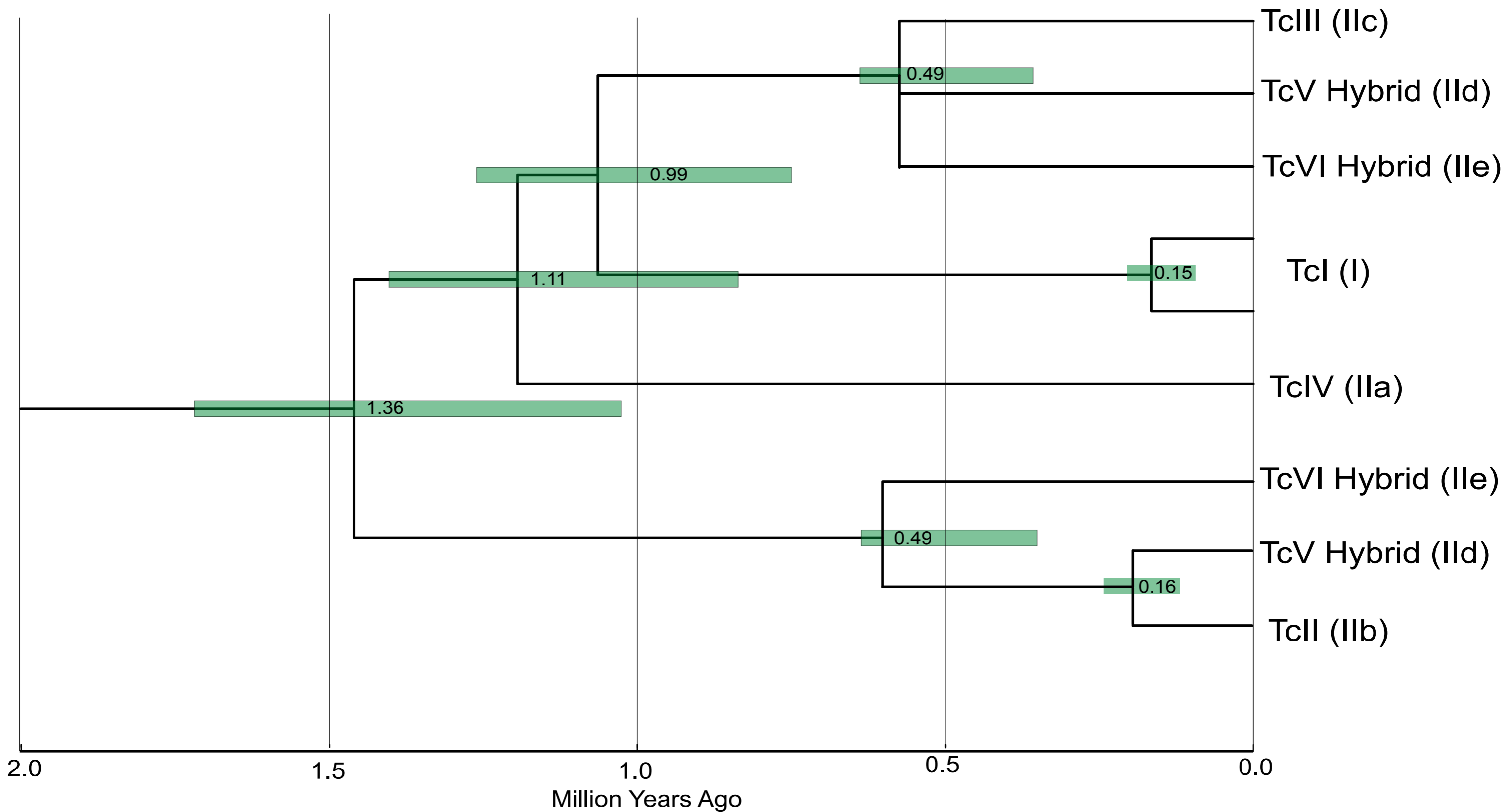

Supplement: Figure S2 — Divergence times for main DTU clades of T. cruzi using nuclear loci with the strict clock model. Data set consists of aligned concatenated nuclear loci (22) for which the molecular clock was not rejected (Table S4), and had a homolog in T. brucei. Codes (Strains): TcI (SO34 cl4 & SC13), TcIV (EP 255), TcII (CBB cl3), TcIII (M6241 cl6), TcV Hybrid (SO3 cl5), TcVI Hybrid (CL Brener) (See Table 1). Scale bar in millions of years ago. (PDF) [file pntd.0001272.s002.pdf]
