# Supplementary material for: Analyses of 32 Loci Clarify Phylogenetic Relationships among Trypanosoma cruzi Lineages and Support a Single Hybridization prior to Human Contact
Source: PLoS Negl Trop Dis. 2011 Aug 2;5(8):e1272. doi: 10.1371/journal.pntd.0001272 (PMC3149036; doi:10.1371/journal.pntd.0001272)
Supplement: Table S1 — Additional Trypanosoma cruzi strains used in this study. (DOC) [file pntd.0001272.s003.doc]

**Table S1.** Additional *Trypanosoma cruzi* strains used for some of the loci.

| **Strains** | **DTU** ^a^ | **Zymodeme** ^b^ | **Isoenzyme**  **types** ^c^ | **1999**  **classification** ^d^ | **2009**  **nomenclature** ^e^ |
| --- | --- | --- | --- | --- | --- |
| 133 79 cl7, CUICA cl1, TEH cl2 cl92, CEPA EP, Vin C6, FLORIDA C16, X10 cl1, SABP3, A80, A92, MA-V, OPS21 cl11, Esquilo cl1, CUTIA cl1, V121, 2679, P209 cl1, 85/818, P0AC | I | Z1 | 12,17,19,20 | TcI | TcI |
| TU18 cl2, ESMERALDO cl3, X-300, MSC2, MCV, MVB cl8 | IIb | Z2 | 30,32 | TcII | TcII |
| M5631 cl5, CM 17, X110/8, X9/3, X109/2 | IIc | Z2 | 36 | TcII | TcIII |
| CANIII cl1 | IIa | Z3 | 27 | TcII | TcIV |
| EPP, PSC-O, 86-1 | IId | Z2 | 39 | TcII | TcV |
| P251, TULAHUEN cl2, P63 cl1, 86/2036, VMV4 | IIe | Z2 | 43 | TcII | TcVI |

^a^ Discrete typing unit (DTU) [8], ^b^ [4], ^c^ [6], ^d^ [30], ^e^ [32].
