# Supplementary material for: Analyses of 32 Loci Clarify Phylogenetic Relationships among Trypanosoma cruzi Lineages and Support a Single Hybridization prior to Human Contact
Source: PLoS Negl Trop Dis. 2011 Aug 2;5(8):e1272. doi: 10.1371/journal.pntd.0001272 (PMC3149036; doi:10.1371/journal.pntd.0001272)
Supplement: Table S2 — Amplified loci and PCR primers. (DOC) [file pntd.0001272.s004.doc]

**Table S2.** Amplified nuclear loci and PCR primers. Locus ID of *T. brucei* homolog was obtained from tritrypdb.org.

| **Locus ID** | **Sequenced (bp)** | **Predicted function** | **Chromosome**  **location** | **Location in Chr.**  **Gene length** | **Primer sequence (5’-3’)** | **Homologue in**  ***T. brucei*** |
| --- | --- | --- | --- | --- | --- | --- |
| HSP70 | 508 | Intergenic region | Chr 32 | 699686 - 700540 (-)  Length: 855 bp | AGGGTGATCAGCAGAAGCAG  CGCAAACGACGAGCGAACAT | N/A |
| Tc00.1047053503885.80 | 946 | Not known | Chr 26 | 163788 - 164993 (+)  Length: 1206 bp | ACAATCGATGTGCTTGACGA  CAGTACGAGCCCGAGACATC | Tb927.8.6320 |
| Tc00.1047053503891.50 | 813 | Not known | Chr 20 | 75320 - 76489 (-)  Length: 1170 bp | CACACCGTCTCTTCCCACTT  CCGCTATGTCCATTTCACCT | Tb927.10.4750 |
| Tc00.1047053503909.76 | 614 | Ferric reductase transmembrane protein | Chr 32 | 556434 - 557156 (+)  Length: 723 bp | GGAGCAACCGCATCTTTTAC  TACGATACGCCAAAGTACGC | Tb927.6.3320 |
| Tc00.1047053504013.40 | 805 | Serine acetyltransferase | Chr34 | 465693 - 466718 (-)  Length: 1026 bp | TCGAAGTCATTCGGAAGTCA  TGGCGTAGATGGTCACTCTG | Not found |
| Tc00.1047053504045.100 | 886 | Not known | Chr 40 | 1854961 - 1856415 (-)  Length: 1455 bp | GCAGCGGCAGTTCTTTATTC  AGCCTTTCGCTCATTCTCAA | Tb927_03_v4 |
| Tc00.1047053504057.80 | 858 | Not known | Chr 34 | 417310 - 418677 (-)  Length: 1368 bp | ATTACGCCCTTTGTCCAGTG  GACGGGACAAGAAAGATCCA | Tb927.4.1590 |
| Tc00.1047053504059.20 | 896 | Endomembrane protein, putative | Chr 14 | 465730 - 467526 (-)  Length: 1797 bp | TGAGGGAGGAATTGGTTGAG  TGCACCAAATCCAAATGAAA | Tb11.02.0960 |
| Tc00.1047053506247.200 | 920 | Beta-adaptin, role inferred from homology | Chr 37 | 133811 - 136708 (+)  Length: 2898 bp | TGAGTCATTACAGCGCAAGG  TCTTCACTGGCTTCCTCGTT | Tb927.10.8040 |
| Tc00.1047053506525.150 | 821 | Not known | Chr 40 | 593462 - 594415 (+)  Length: 954 bp | GCCGCTGATACGGACAAG  CAAGTCAGAGACGGTGTCAGG | Tb927.10.14310 |
| Tc00.1047053506529.310 | 727 | Not known | Chr 6 | 97318 - 98676 (-)Length: 1359 bp | TTCTTTCAGGCTGCGATTTTCGCTGTTTGGCTCATTTCTT | Tb927.1.4220 |
| Tc00.1047053506739.20 | 810 | Not known | Chr 3 | 25655 - 27589 (-)  Length: 1935 bp | AGCTAAGCACACTCGCCAAT  CAATCTCTCGAGCCGTTCTC | Tb927.5.1500 |
| Tc00.1047053507801.70 | 677 | Protein kinase | Chr 23 | 535126 - 535959 (+)  Length: 834 bp | AAAGAGTTGCCGTCAAGGTG  CATGGGTGTTCCAATGACTG | Tb927.2.5230 |
| Tc00.1047053508153.540 | 774 | Not known | Chr 36 | 699363 - 700391 (+)  Length: 1029 bp | GCATTCGAGGAGAGAACGAG  GCGCTCTCAGAAGCAAAGTT | Tb927.3.3060 |
| Tc00.1047053508461.80 | 838 | Prostaglandin F2 alpha synthase | Chr 39 | 1187987 - 1189126 (-) Length: 1140 bp | TCGGATTCCTGCCTATTTTG  TGTTTGCATTTTCCCACTGA | Not found |
| Tc00.1047053508719.70 | 709 | Not known | Chr 37 | 375185 - 376402 (+)  Length: 1218 bp | AAAATTGTCCATGCGAGTCC  CACCAAATCCTTGCGTTTCT | Tb927.10.8940 |
| Tc00.1047053509007.30 | 815 | Not known | Chr 31 | 573767 - 574690 (+)  Length: 924 bp | CTTCCACGATGCGCTACAG  GGAGCACACAATCTCCTTCC | Tb927.8.7810 |
| Tc00.1047053509105.70 | 897 | thiol-dependent reductase 1 | Chr 37 | 769449 - 770786 (-)  Length: 1338 bp | ATGGTCGTTCCATTTCTTGC  TAAGCCACTCCTTGGTGGAC | Not found |
| Tc00.1047053509561.20 | 880 | Flagellum-adhesion glycoprotein | Chr 12 | 285842 - 287581 (-)  Length: 1740 bp | CACCCTTGCCGGTAGTAAAA  TCTATCTGGCGGAAATACGG | Tb927.8.4110 |
| Tc00.1047053509967.50 | 595 | Not known | Chr 10 | 184622 - 185329 (+)  Length: 708 bp | TCTTGACATCGGGAGTAGCC  ACACCAAAACACTTGGCACA | Tb927.10.1240 |
| Tc00.1047053510101.480 | 829 | Not known | Chr 27 | 190063 - 191427 (-)  Length: 1365 bp | CAGCGCATTGAAGATTGTGT  TGCATCAACTGAAGGTCTGC | Tb11.02.4410 |
| Tc00.1047053510123.24 | 880 | Not known | Chr 20 | 372476 - 373429 (+)  Length: 954 bp | ATGCATGCAGGAACACAAAT  TTCACTCGTACTGGGTGTCG | Tb927.10.12030 |
| Tc00.1047053510131.90 | 936 | Not known | Chr 30 | 340360 - 342003 (+)  Length: 1644 bp | ATCGACATGGAACTCGAACC  GTACTCCTCCGTGACCCAAA | Not found |
| Tc00.1047053510765.50 | 817 | Not known | Chr 39 | 1780396 - 1781763 (+)  Length: 1368 bp | TTGTGTTGCTAAGGCACTGG  AATGAGACCCTCGCAAAGAA | Tb11.12.0004 |
| Tc00.1047053510877.190 | 453 | Not known | Chr 34 | 493531 - 494328 (-)  Length: 798 bp | TCTGGACTCGTACGTCTACCC  GGGACGTCCGTTCACGTAT | Tb927.4.1910 |
| Tc00.1047053510889.210 | 693 | Not known | Chr 6 | 154383 - 156290 (-)  Length: 1908 bp | ATGGAATTGGAGCAAGAACG  GGTAAAAGCCGCATCAGAAA | Tb927.1.3840 |
| Tc00.1047053510889.310 | 763 | Not known | Chr 6 | 193929 - 196025 (+)  Length: 2097 bp | GTTTGGGCAACACGAAAGAT  TGATGTCTGCTTGGAACCTG | Tb927.1.3450 |
| Tc00.1047053511153.124 | 513 | Not known | Chr 27 | 412720 - 413271 (+) Length: 552 bp | CGTCTTTGGGATTTCTGTCCGGTGTCAGGCTGGTC CTCT | Not found |
| Tc00.1047053511529.200 | 667 | Not known | Chr 35 | 170438 - 171232 (-)  Length: 795 bp | GTGAGGCGCGAAGAAAATAC  TACGAAACGTTGCCGTCAG | Not found |
| Tc00.1047053503555.30 (TR) ^a^ | 1290 | Trypanothione reductase | Chr 37 | 713055 - 714533 (-) Length: 1479 bp | ACTGGAGGCTGCTTGGAACGC  GGATGCACACCRATRGTGTTGT | Tb927.10.10390 |
| Tc00.1047053509153.90 (DHFR-TS) ^a^ | 1473 | Dihydrofolate reductase-thymidylate synthase | Chr 27 | 718463 - 720028 (+)  Length: 1566 bp | CGCTGTTTAAGATCCGNATGCC  CGCATAGTCAATGACCTCCATGTC | Tb927.7.5480 |

**^a^** Sequence data from Machado & Ayala [10]
