# Supplementary material for: Analyses of 32 Loci Clarify Phylogenetic Relationships among Trypanosoma cruzi Lineages and Support a Single Hybridization prior to Human Contact
Source: PLoS Negl Trop Dis. 2011 Aug 2;5(8):e1272. doi: 10.1371/journal.pntd.0001272 (PMC3149036; doi:10.1371/journal.pntd.0001272)
Supplement: Table S3 — Results of the Shimodaira-Hasegawa tests. (DOC) [file pntd.0001272.s005.doc]

**Table S3.** Results of the Shimodaira-Hasegawa tests.

| **Gene ID** | **ML tree ^a^** | **TcI-TcII  ^a^** | **S-H Best tree  ^b^** |
| --- | --- | --- | --- |
| COII-ND1 | 3185.44688 | 3203.15949 | ML tree* |
| Tc00.1047053503555.30 | 2435.87661 | 2670.43397 | ML tree** |
| Tc00.1047053509153.90 | 2907.60509 | 3622.74370 | ML tree** |
| HSP70 | 1365.70184 | 1631.03893 | ML tree** |
| Tc00.1047053503885.80 | 2012.54516 | 2277.85677 | ML tree** |
| Tc00.1047053503891.50 | 1859.67704 | 2020.17966 | ML tree** |
| Tc00.1047053503909.76 | 1312.97442 | 1441.75777 | ML tree** |
| Tc00.1047053504013.40 | 1738.01299 | 1986.07561 | ML tree** |
| Tc00.1047053504045.100 | 1895.77443 | 1959.24750 | ML tree* |
| Tc00.1047053504057.80 | 1616.35925 | 1676.26866 | ML tree* |
| Tc00.1047053504059.20 | 1879.71822 | 2021.47943 | ML tree** |
| Tc00.1047053506247.200 | 1778.40746 | 1825.83641 | ML tree** |
| Tc00.1047053506525.150 | 1628.37847 | 1809.03975 | ML tree** |
| Tc00.1047053506529.310 | 1560.80452 | 1707.20155 | ML tree** |
| Tc00.1047053506739.20 | 1812.77614 | 1976.33301 | ML tree** |
| Tc00.1047053507801.70 | 1416.29930 | 1521.99612 | ML tree** |
| Tc00.1047053508153.540 | 1584.79227 | 1701.44882 | ML tree* |
| Tc00.1047053508461.80 | 1264.19040 | 1336.38017 | ML tree* |
| Tc00.1047053508719.70 | 1367.05726 | 1493.09884 | ML tree* |
| Tc00.1047053509007.30 | 1721.93006 | 1894.18590 | ML tree** |
| Tc00.1047053509105.70 | 1818.85886 | 2086.09222 | ML tree** |
| Tc00.1047053509561.20 | 1908.84944 | 2141.94614 | ML tree** |
| Tc00.1047053509967.50 | 1304.46342 | 1380.39366 | ML tree* |
| Tc00.1047053510101.480 | 1655.11244 | 1775.00741 | ML tree** |
| Tc00.1047053510123.24 | 2074.22941 | 2285.24245 | ML tree** |
| Tc00.1047053510131.90 | 2029.45289 | 2168.68034 | ML tree** |
| Tc00.1047053510765.50 | 1752.33457 | 1965.21006 | ML tree** |
| Tc00.1047053510877.190 | 973.63644 | 1005.17473 | ML tree* |
| Tc00.1047053510889.210 | 1500.68706 | 1723.67076 | ML tree** |
| Tc00.1047053510889.310 | 1451.22617 | 1670.24522 | ML tree** |
| Tc00.1047053511153.124 | 1151.05222 | 1302.29934 | ML tree** |
| Tc00.1047053511529.200 | 1376.77905 | 1586.65525 | ML tree** |

**^a^** –ln likelihood value for the phylogeny reconstructed under the Maximum Likelihood criteria with no topological constraints or when TcI and TcII are constrained to be reciprocally monophyletic.

**^b^** The most plausible tree according to the Shimodaira-Hasegawa test. *significant at p≤0.05, **significant at p≤0.001.
