# Supplementary material for: Analyses of 32 Loci Clarify Phylogenetic Relationships among Trypanosoma cruzi Lineages and Support a Single Hybridization prior to Human Contact
Source: PLoS Negl Trop Dis. 2011 Aug 2;5(8):e1272. doi: 10.1371/journal.pntd.0001272 (PMC3149036; doi:10.1371/journal.pntd.0001272)
Supplement: Table S4 — LRT of Molecular clock on genes that had a homolog in T. brucei. (DOC) [file pntd.0001272.s006.doc]

**Table S4**. LRT of Molecular clock on genes that had a homolog in *T. brucei*

| **Gene** | **N^a^** | **-ln L**  **Enforced clock** | **-ln L**  **No clock** | **LRT^b^** |
| --- | --- | --- | --- | --- |
| COII-ND1 | 9 | 3176.531 | 3171.313 | 10.436 (0.16) |
| TR | 12 | 3541.111 | 3536.922 | 8.37 (0.59) |
| DHFR-TS | 12 | 2367.433 | 2355.957 | 22.95 **(0.01)*** |
| Tc00.1047053503885.80 | 13 | 3086.302 | 3079.941 | 12.72 (0.31) |
| Tc00.1047053503891.50 | 11 | 3154.729 | 3147.585 | 14.28 (0.11) |
| Tc00.1047053504045.100 | 11 | 2918.118 | 2914.197 | 7.84 (0.55) |
| Tc00.1047053504057.80 | 11 | 2470.922 | 2465.348 | 11.14 (0.26) |
| Tc00.1047053504059.20 | 14 | 2932.983 | 2919.348 | 27.27 **(0.007)*** |
| Tc00.1047053506247.200 | 11 | 2679.311 | 2676.382 | 5.85 (0.75) |
| Tc00.1047053506525.150 | 14 | 2748.648 | 2743.028 | 11.24 (0.5) |
| Tc00.1047053506529.310 | 14 | 2296.608 | 2286.840 | 19.53 (0.07) |
| Tc00.1047053506739.20 | 9 | 2696.520 | 2691.508 | 10.02 (0.18) |
| Tc00.1047053507801.70 | 13 | 4355.910 | 4350.086 | 11.64 (0.39) |
| Tc00.1047053508153.540 | 14 | 2793.654 | 2784.699 | 17.91 (0.11) |
| Tc00.1047053508719.70 | 14 | 2152.143 | 2147.313 | 9.66 (0.64) |
| Tc00.1047053509007.30 | 14 | 2701.612 | 2696.047 | 11.13 (0.51) |
| Tc00.1047053509561.20 | 13 | 2980.772 | 2970.446 | 20.65 **(0.03)*** |
| Tc00.1047053509967.50 | 12 | 2287.766 | 2283.898 | 7.73 (0.65) |
| Tc00.1047053510101.480 | 13 | 2510.700 | 2503.038 | 15.32 (0.16) |
| Tc00.1047053510123.24 | 13 | 3060.619 | 3057.453 | 6.33 (0.85) |
| Tc00.1047053510765.50 | 14 | 2976.456 | 2971.093 | 10.72 (0.55) |
| Tc00.1047053510877.190 | 9 | 1957.833 | 1955.794 | 4.07 (0.77) |
| Tc00.1047053510889.210 | 14 | 2150.871 | 2145.585 | 10.57 (0.56) |
| Tc00.1047053510889.310 | 12 | 2383.305 | 2379.224 | 8.162 (0.61) |

^a^ Number of sequences.

^b^ Likelihood Ratio Test (p value; estimated value from chi-square distribution with df=s-2, where s is the number of taxa). * Molecular clock rejected.
